# Echinatin attenuates acute lung injury and inflammatory responses via TAK1-MAPK/NF-κB and Keap1-Nrf2-HO-1 signaling pathways in macrophages

**DOI:** 10.1371/journal.pone.0303556

**Published:** 2024-05-16

**Authors:** Liuling Luo, Huan Wang, Jinrui Xiong, Xiaorui Chen, Xiaofei Shen, Hai Zhang

**Affiliations:** 1 State Key Laboratory of Southwestern Chinese Medicine Resources, School of Pharmacy/School of Modern Chinese Medicine Industry, Chengdu University of Traditional Chinese Medicine, Chengdu, China; 2 Hospital of Chengdu University of Traditional Chinese Medicine, Chengdu, China; Taylor’s University, MALAYSIA

## Abstract

Echinatin is an active ingredient in licorice, a traditional Chinese medicine used in the treatment of inflammatory disorders. However, the protective effect and underlying mechanism of echinatin against acute lung injury (ALI) is still unclear. Herein, we aimed to explore echinatin-mediated anti-inflammatory effects on lipopolysaccharide (LPS)-stimulated ALI and its molecular mechanisms in macrophages. *In vitro*, echinatin markedly decreased the levels of nitric oxide (NO) and prostaglandin E2 (PGE_2_) in LPS-stimulated murine MH-S alveolar macrophages and RAW264.7 macrophages by suppressing inducible nitric oxide synthase and cyclooxygenase-2 (COX-2) expression. Furthermore, echinatin reduced LPS-induced mRNA expression and release of interleukin-1β (IL-1β) and IL-6 in RAW264.7 cells. Western blotting and CETSA showed that echinatin repressed LPS-induced activation of mitogen-activated protein kinase (MAPK) and nuclear factor-kappa B (NF-κB) pathways through targeting transforming growth factor-beta-activated kinase 1 (TAK1). Furthermore, echinatin directly interacted with Kelch-like ECH-associated protein 1 (Keap1) and activated the nuclear factor erythroid 2-related factor 2 (Nrf2) pathway to enhance heme oxygenase-1 (HO-1) expression. *In vivo*, echinatin ameliorated LPS-induced lung inflammatory injury, and reduced production of IL-1β and IL-6. These findings demonstrated that echinatin exerted anti-inflammatory effects *in vitro* and *in vivo*, via blocking the TAK1-MAPK/NF-κB pathway and activating the Keap1-Nrf2-HO-1 pathway.

## Introduction

Acute lung injury (ALI) is a chronic clinical syndrome caused by serious inflammatory damage that could progress to life-threatening acute respiratory distress syndrome (ARDS) unless promptly treated [1, 2]. The pathology of ALI is characterized by the activation and infiltration of inflammatory cells, respiratory distress, and diffuse pulmonary edema [3, 4]. ALI is generally caused by various internal and external factors including sepsis, pneumonia, ischemia reperfusion, drug toxicity, and trauma [1, 5]. The current treatment of ALI focuses on reducing inflammation and suppressing respiratory failure with drugs such as dexamethasone, prednisolone, and prednisone [6, 7]. These drug treatments can cause various adverse effects, including coagulation disorders and osteoporosis [6]; therefore, novel drugs for treating ALI are urgently required.

Macrophages play a significant role in the onset, maintenance, and regression of inflammation [8, 9]. Toll-like receptor 4 (TLR4) is a pattern recognition receptor (PRR) capable of recognizing evolutionarily conserved components of microorganisms, including bacteria, collectively called pathogen-associated molecular patterns (PAMPs) [10]. Increasing evidence has revealed that TLR4 can be activated in lipopolysaccharide (LPS)-induced macrophages, followed by stimulating the mitogen-activated protein kinase (MAPK)- and nuclear factor-kappa B (NF-κB)-mediated inflammatory responses, inducing the expression of several pro-inflammatory cytokines and enzymes [11, 12]. Several inflammatory mediators and cytokines, including nitric oxide (NO), prostaglandin E2 (PGE_2_), interleukin-1 beta (IL-1β), and IL-6 can induce oxidative stress, which cumulatively cause lung tissue injury [13–15]. Therefore, repressing the biological functions of macrophages via blocking MAPK and NF-κB pathways activation may be a prospective strategy for the therapy of ALI. Simultaneously, the inflammatory response can cause oxidative stress, further leading to tissue damage. Nuclear factor erythroid 2-related factor 2 (Nrf2) is a central pathway regulating oxidative stress and suppressing the overproduction of reactive oxygen species by inducing heme oxygenase-1 (HO-1) expression [16]. Therefore, activating this pathway also represses oxidative stress and controls acute inflammatory responses.

Licorice, the roots and rhizomes of several *Glycyrrhiza* species (Leguminosae), has been used as a traditional remedy for asthma and inflammatory conditions in China [17]. Echinatin is a characteristic chalcone present in licorice, exhibiting various anti-inflammatory and antioxidative therapeutic effects [18]. Echinatin was reported to exhibit high reactive oxygen scavenging and potential antioxidant properties, and it also inhibited NO, PGE_2_, IL-6, and reactive oxygen species production [19] in LPS-stimulated RAW264.7 macrophages [18, 20, 21]. Echinatin treatment markedly repressed the activation of NLRP3 inflammasome and attenuated LPS-stimulated septic shock as well as dextran sodium sulfate stimulation colitis *in vivo* [22]. However, the pharmacological activities, underlying mechanisms, and functional targets of echinatin in ALI have not been reported. Therefore, in the present study, we aimed to examine echinatin-mediated anti-inflammatory effects and underlying mechanisms in LPS-induced ALI mice and macrophages.

## Materials and methods

### Chemicals and reagents

**Fig 1:**
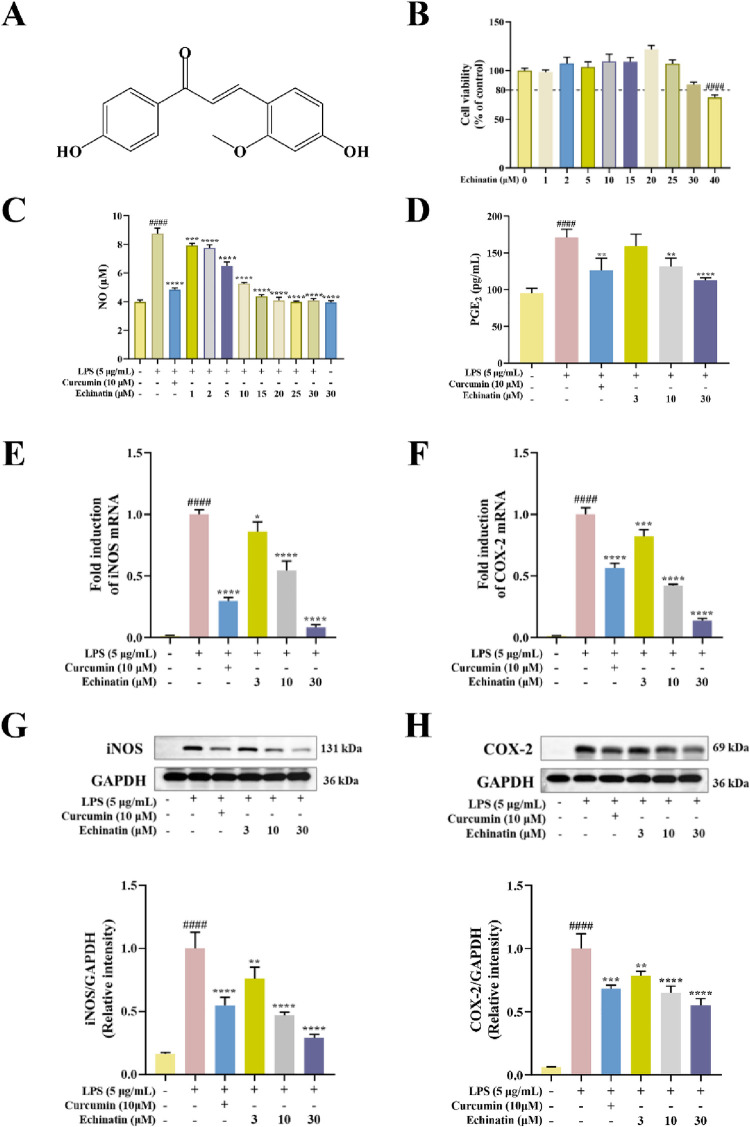
Echinatin inhibits LPS-induced inflammatory responses in MH-S cells. **(A)** The chemical structure of echinatin. **(B)** MH-S cells were treated with different concentrations of echinatin for 24 h. The cell viability of these MH-S cells was measured by CCK-8 assay. After echinatin pretreatment for 2 h, the MH-S cells were cultured with LPS (5 μg/mL) for an additional 24 h. **(C)** The release of NO was measured by Griess reagent. **(D)** The effect of echinatin on PGE_2_ production was measured by ELISA kit. The gene expression of iNOS **(E)** and COX-2 **(F)** in MH-S cells was measured by qRT-PCR using GAPDH as the internal control. The protein levels of iNOS **(G)** and COX-2 **(H)** were measured by Western blotting. GAPDH is used as an internal reference. Data are represented as mean ± SD. ^**#**^ *p* < 0.05, ^**##**^ *p* < 0.01, ^**###**^ *p* < 0.001, ^**####**^ *p* < 0.0001 *vs*. the control group. ^*^ *p* < 0.05, ^**^ *p* < 0.01, ^***^ *p* < 0.001, ^****^ *p* < 0.0001 *vs*. the LPS group.

Echinatin (purity > 98%, Fig 1A, compound CID: 6442675) and Curcumin (purity > 98%, compound CID: 969516) were both obtained from Sichuan ChemConst Biotechnology Co., Ltd (Chengdu, China). LPS was extracted from *Escherichia coli* O55:B5 and was procured from Sigma-Aldrich (St. Louis, Mo, USA). SP600125 (JNK inhibitor) and SB203580 (p38 inhibitor) were obtained from Beyotime (Shanghai, China). BAY 11–7082 (BAY, NF-κB inhibitor) and PD98059 (ERK inhibitor) were obtained from MedChemExpress (Monmouth Junction, NJ, USA). 5Z-7-Oxozeaenol (OZL, TAK1 inhibitor) was obtained from APExBIO Technology (Houston, USA). Antibodies against COX-2, GAPDH, iNOS, PARP1, β-actin, tubulin, and horseradish peroxidase-conjugated AffiniPure goat antibody were obtained from the ProteinTech Group (Wuhan, China). Primary antibodies against extracellular regulated protein kinase (ERK), phosphorylated ERK (p-ERK), c-Jun N-terminal kinase (JNK), p-JNK, p38, p-p38, transforming growth factor-β activated kinase-1 (TAK1), inhibitor of kappa B kinase (IKK), p-IKK, inhibitor of kappa B (IκBα), p-IκBα, and p65 were purchased from Beyotime (Shanghai, China). Antibodies against kelch-like ECH-associated protein 1 (Keap1), Nrf2, HO-1 and p-TAK1 were obtained from Affinity Biosciences (Cincinnati, OH, USA). PCR primers (S1 Table) including iNOS, COX-2, IL-1β, IL-6, and GAPDH were obtained from Sangon Biotech (Shanghai, China).

### Cell culture

Murine RAW264.7 peritoneal macrophages were obtained from Beyotime (Shanghai, China). The cells were cultured in high-glucose Dulbecco’s modified eagle medium (DMEM; Gibco, USA), containing 10% fetal bovine serum (FBS; 55°C inactivation, PAN biotech, Germany) and cultured at 37°C, 95% humidified atmosphere, and 5% CO_2_. Murine MH-S alveolar macrophages were purchased from Procell (Wuhan, China). The cells were cultured in MH-S cell-specific medium (Procell, Wuhan, China), containing RPMI-1640 medium, 10% FBS, 1% penicillin/streptomycin, and 0.05 mM of β-mercaptoethanol in a 37°C, 5% CO2 and 95% humidified atmosphere.

### Cell viability assay

Cell viability was assessed using the cell counting kit-8 (CCK-8) assay after treatment with the samples. The RAW264.7 cells (1 × 10^4^ cells/well) and MH-S cells (2 × 10^4^ cells/well) were seeded into 96-well plates overnight and then treated with diverse echinatin concentrations for 24 h. Next 10 μL of CCK-8 solution (Boster, Wuhan, China) was added to each well and incubated for another 1 h at 37°C. The absorbance at 450 nm was determined using a multifunctional microplate reader (Molecular Devices, USA).

### Determination of NO production

RAW264.7 cells (1 × 10^4^ cells/well) or MH-S cells (2 × 10^4^ cells/well) were seeded in a 96-well plate overnight and then pretreated with curcumin (positive control) and various concentrations of echinatin for 2 h. Next, RAW264.7 cells were stimulated with LPS (0.5 μg/mL) and MH-S cells with 5 μg/mL LPS, respectively. After coculturing for 24 h, the levels of NO in the cell culture were detected using the Griess reagent (Beyotime, Shanghai, China).

### Measurement of inflammatory mediators and cytokines

After being treated with echinatin and LPS, the cell culture medium was harvested and centrifuged for 3 min at 4°C (1000 g). The supernatants were to detect the levels of PGE_2_, IL-1β, and IL-6 using enzyme-linked immunosorbent assay kits (R&D Systems, Minneapolis, USA) according to the manufacturer’s guidelines. The concentrations of PGE_2_, IL-1β, and IL-6 were calculated using the standard curves.

### Quantitative real-time polymerase chain reaction

The mRNA levels of iNOS, COX-2, IL-1β, and IL-6 were detected using qRT-PCR. RAW264.7 cells were plated in a 6-well plate at a density of 2 × 10^6^ cells/well, incubated overnight, and then treated with different echinatin concentrations (3, 10, and 30 μM) and Curcumin (20 μM) for 2 h, followed by LPS treatment (0.5 μg/mL) for additional 24 h. Total RNA isolation Kit (Foregene, Chengdu, China) was used for total RNA extraction. Subsequently, the total RNAs were reverse-transcribed to cDNAs using All-in-One cDNA Synthesis Super Mix Kit (GeneCopoeia, Guangzhou, China) based on the manufacturer’s instructions. qRT-PCR was performed using a qTOWER^3^ G Real-Time fluorescence quantitative PCR instrument (Analytik Jena, Thuringia, Germany). Relative expression levels of the target genes were calculated by using the 2^-ΔΔCt^ method according to the manufacturer′s instructions considering the GAPDH gene as a normalization.

### Western blot analysis

Total protein was extracted from macrophages using pre-cold radio immune precipitation assay buffer (Boster, Wuhan, China) in the presence of 1% protease and phosphatase inhibitors (Boster, Wuhan, China). The cell lysates were centrifuged at 12,000 g at 4°C for 10 min, mixed with a loading buffer, and boiled for 5 min at 98°C. The obtained supernatants were separated using sodium dodecyl sulfate-polyacrylamide gel electrophoresis. The proteins were subsequently blotted onto the poly (vinylidene fluoride) membrane (Millipore, Bedford, USA). The membranes were then blocked with 5% bovine serum albumin in TBST buffer and incubated with the following primary antibodies overnight at 4°C. After the solutions were washed three times with TBST buffer, the membranes were incubated with (HRP)-conjugated secondary antibodies (1:5000, Beyotime) for 1 h at room temperature and then washed with TBST for three times. The blotting signals were detected using an enhanced chemiluminescence reagent (Beyotime, Shanghai, China), and quantified using ImageJ.

### Nuclear and cytoplasmic protein extraction

After being treated with echinatin for 2 h and treated with LPS for 30 min, cells were harvested via centrifugation (1500 *g* for 3 min) at 4°C. The cytoplasmic and nuclear proteins were separated according to the manufacturer’s instructions provided in Nuclear and Cytoplasmic Protein Extraction Kit (Beyotime, Shanghai, China). The cytoplasmic and nuclear distributions of p65 and Nrf2 were detected using Western blotting. The GAPDH and PARP1 were used as the loading controls of cytoplasmic and nuclear proteins, respectively.

### Cellular thermal shift assay (CETSA)

To detect the thermal stability of the binding between echinatin and potential protein targets, CETSA was employed. RAW264.7 cells were inoculated in 100-mm culture dishes, and the experiment was performed when the cells reached 80% confluence. The cells were collected and resuspended in 1 mL of phosphate-buffered saline containing 1% protease inhibitor, and the solution was subjected to freeze-thawing using liquid nitrogen (3 times). The suspension was centrifuged at 12000 *g* at 4°C for 10 min and equal amounts were incubated with echinatin (100 μM) or dimethyl sulfoxide (DMSO) at 37°C for 30 min. Aliquots of the mixtures were added to 14 PCR tubes and heated at 45°C, 48°C, 51°C, 54°C, 57°C, 60°C, and 63°C for 3 min in a thermal cycler. The samples were then centrifuged at 12000 g for 10 min at 4°C, mixed with 5 × loading buffer, boiled at 100°C for 5 min, and subjected to Western blotting.

### Molecular docking

Molecular docking simulation was used to detect the binding abilities of echinatin to target proteins Keap1 and TAK1. The crystal structures of human Keap1 (PDB ID: 6LRZ) and TAK1 (PDB ID: 4O91) were obtained from the Protein Data Bank database (http://www.rcsb.org/) in PDB format and preformed using the AutoDock Vina program, which included the addition of polar hydrogen and removal of water and ligands. The chemical structure of echinatin was determined using the PubChem database. AutoDock.exe was used for molecular docking and analysis of docking results. The docking interaction between the key target proteins and the molecular ligands was displayed using PyMOL software in a 3D format.

### Animals and treatment

Male ICR mice (4–6 weeks old; 20–22 g, SPF grade) were provided by Beijing Hua-Fu-Kang Biotechnology (Beijing, China, SCXK (Beijing) 2019–0008). The mice were housed under a 12-h light/dark cycle along with suitable temperature and humidity conditions. The mice had free access to standard food and tap water. All experimental animals were adapted to the aforementioned conditions at least 7 days before the experiments. All experiments were approved by the Committee of Chengdu University of Traditional Chinese Medicine and Institutional Animal Care (Protocol Number: 2022–27). All surgery was performed under sodium pentobarbital anesthesia, and all efforts were made to minimize suffering.

The mice were divided into the following seven groups (n = 6): the control group, LPS group, dexamethasone-treated (DEX, 0.1 mg/kg) + LPS group, echinatin (10 mg/kg) + LPS group, echinatin (20 mg/kg) + LPS group, and echinatin (50 mg/kg) + LPS group. Mice were administered intragastrically with DEX or echinatin for 7 consecutive days (1 time/day). The control group and LPS group were administered with 0.5% sodium carboxymethyl cellulose. After 2 h of the last administration, mice were intravenously injected with LPS (10 mg/kg) via tail vein.

### Cytokine detection and histological examination

After being injected with LPS for 12h, mice were anesthetized with pentobarbital sodium (25 mg/kg). Blood samples were collected from the orbital venous plexus. The serum was harvested via centrifuging at 1,000 g for 5 min. The levels of IL-1β and IL-6 were measured using ELISA kits. Next, mice were sacrificed by dislocation. The lung tissues were removed, and were fixed with 4% paraformaldehyde for 24 h and dehydrated in series concentrations of ethanol. The lung tissue samples were processed into 5-μm slices for hematoxylin and eosin (H&E) staining. The H&E staining was used to investigate the protective role of echinatin on pulmonary inflammatory impairment. The sections were dewaxed with xylene and mixed with hematoxylin for 5 min before successively incubating with 1% hydrochloric acid alcohol and 1% ammonia. Then, eosin was added for additional staining for 5 min. The sections were observed under a fluorescence microscope (Nikon, Tokyo, Japan). Pathological scoring of lung tissues was assessed by two experienced pathologists who were unaware of the experimental treatment, and the severity of lung injury was scored on the basis of neutrophil infiltration, alveolar wall thickness and alveolar spacing [23]. Lung injury was scored on the following scale: no injury, 0; mild injury with a small amount of neutrophil infiltration and alveolar wall thickening, 1; moderate injury with a moderate amount of neutrophil infiltration, a medium area of alveolar wall thickening, and widened alveolar septa, 2; and severe injury with a large amount of neutrophil infiltration, a large area of alveolar wall thickening, and widened alveolar septa, 3.

### Statistical analysis

Data are expressed as the mean ± standard deviation. Statistical analysis was performed by one-way analysis of variance (ANOVA). All statistical analysis was performed using GraphPad Prism (Version 8.0.1). The statistical significance between the two groups is indicated above the bar for each Fig. *P* < 0.05 was considered statistically significant.

## Results

### Echinatin inhibited LPS-induced inflammatory responses in MH-S cells

To observe whether echinatin has a toxic effect on MH-S murine alveolar macrophages, we determined the cell viability by using the CCK-8 assay. The results showed that echinatin did not affect the cell viability of MH-S cells at 0–30 μM, but exhibited significant cytotoxicity on MH-S cells at 40 μM (*p* < 0.0001) (Fig 1B).

To further investigate the anti-inflammatory effects of echinatin in MH-S cells, we measured the amount of NO and PGE_2_ in MH-S cells. Curcumin is a natural chalcone compound with proven anti-inflammatory activity, therefore, we used curcumin as a positive control [24–26]. The results showed that echinatin markedly inhibited the production of NO in LPS-stimulated MH-S cells in a dose-dependent manner (Fig 1C). However, 30 μM of echinatin had no effect on the production of NO in MH-S cells without LPS incubation. Similarly, echinatin also remarkedly inhibited LPS-induced release of PGE_2_ in MH-S cells in a dose-dependent manner (Fig 1D). Furthermore, as shown in Fig 1E and 1F, the mRNA level of iNOS was increased 100-fold and that of COX-2 was increased 100-fold compared to the control group, and that after echinatin (30 μM) treatment, the mRNA level of iNOS was decreased 12.5-fold and that of COX-2 was decreased 7.14-fold compared to the LPS group. In addition, we found that the protein levels of iNOS and COX-2 were significantly increased after LPS stimulation in MH-S cells, whereas echinatin (3, 10 and 30 μM) treatment distinctly inhibited LPS-induced expression of iNOS and COX-2 in a dose-dependent manner (Fig 1G and 1H). Taken together, these results indicated that echinatin could inhibit LPS-induced inflammatory responses in MH-S alveolar macrophages.

### Echinatin suppressed LPS-induced inflammatory responses in RAW264.7 cells

**Fig 2:**
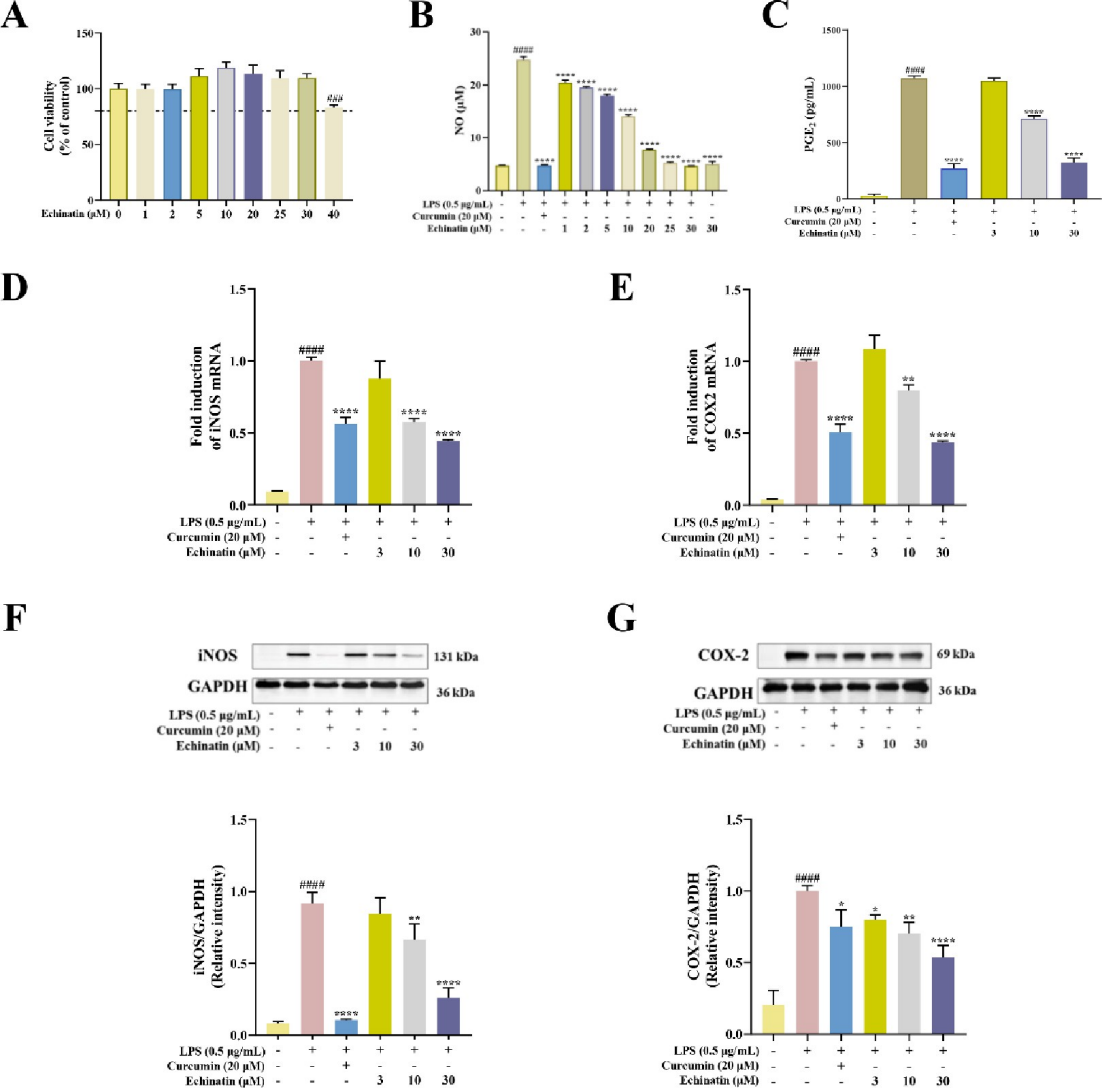
Echinatin suppresses LPS-induced inflammatory response in RAW264.7 cells. **(A)** RAW264.7 cells were treated with different concentrations of echinatin for 24 h. Next, the cell viability of these RAW264.7 cells was detected by CCK-8 assay. After echinatin pretreatment for 2 h, RAW264.7 cells were incubated in the presence of LPS (0.5 μg/mL) for an additional 24 h. The release of NO **(B)** was measured using the Griess reagent. **(C)** The effect of echinatin on PGE_2_ production in LPS-induced RAW264.7 cells. The gene expression of iNOS **(D)** and COX-2 **(E)** was measured by qRT-PCR using GAPDH as the internal control. The protein levels of iNOS **(F)** and COX-2 **(G)** were detected by Western blotting. Data are represented as mean ± SD. ^**#**^ *p* < 0.05, ^**##**^ *p* < 0.01, ^**###**^ *p* < 0.001, ^**####**^ *p* < 0.0001 *vs*. the control group. ^*^ *p* < 0.05, ^**^ *p* < 0.01, ^***^ *p* < 0.001, ^****^ *p* < 0.0001 *vs*. the LPS group.

Next, we utilized another mouse macrophage cell line to further evaluate the anti-inflammatory activity of echinatin. First, the potential cytotoxicity of the drug assessed by CCK8 assay. As shown in Fig 2A, echinatin in the dose range of 0–30 μM had no significant cytotoxicity on RAW264.7 cells, but markedly decreased cell viability at 40 μM. Therefore, the concentrations of echinatin ranging from 0–30 μM were selected for subsequentNO assay. The results suggested that echinatin distinctly suppressed NO production in LPS-stimulated RAW264.7 cells in a dose-dependent manner (Fig 2B). Moreover, 30 μM of echinatin did not affect the baseline level of NO in RAW264.7 cells without LPS stimulation (Fig 2B). In addition, we detected the amount of PGE_2_ in RAW264.7 cells by ELISA kit, and the results showed that compared with the LPS group, the levels of PGE_2_ in the echinatin (10 and 30 μM) treatment groups were significantly decreased (*p* < 0.0001) (Fig 2C).

Subsequently, we measured the mRNA levels of iNOS and COX-2 through qRT-PCR analysis. We found that after LPS stimulation, the mRNA level of iNOS was elevated 11.11-fold and that of COX-2 was elevated 25-fold compared to the control group, and that after echinatin (30 μM) treatment, the mRNA level of iNOS was reduced 2.27-fold and that of COX-2 was reduced 2.27-fold compared to the LPS group (Fig 2D and 2E). Furthermore, we found that echinatin treatment could considerably decrease the protein levels of iNOS and COX-2 in LPS-induced RAW264.7 cells in a dose-dependent manner (Fig 2F and 2G). These results revealed the anti-inflammatory activity of echinatin in LPS-stimulated RAW264.7 macrophages.

### Echinatin downregulated the mRNA expression and release of IL-1β and IL-6 *in vitro*

**Fig 3:**
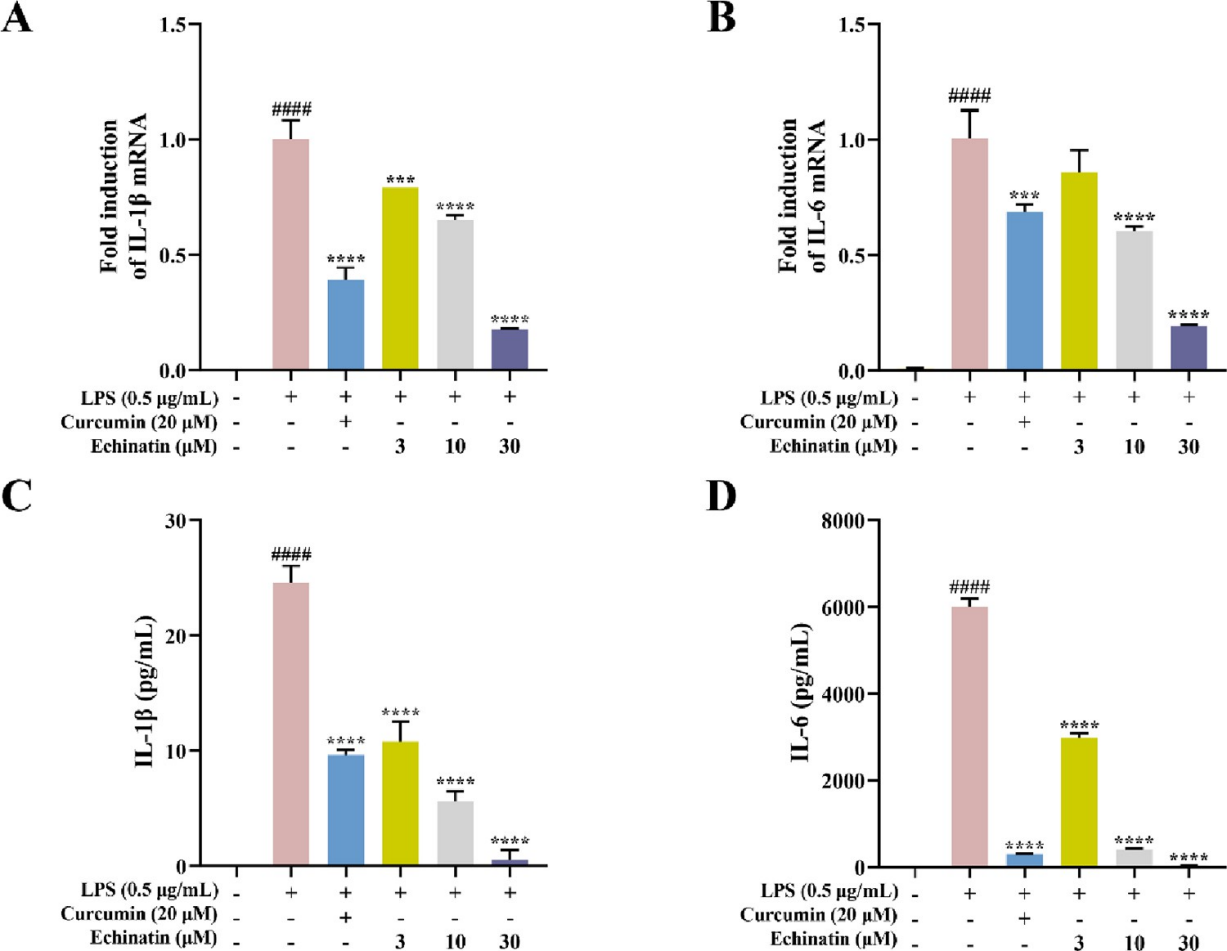
Echinatin decreases the mRNA levels and release of IL-1β and IL-6 in LPS-activated RAW264.7 cells. Cells were pre-treated with different concentrations of echinatin or curcumin for 2 h and treated with LPS (0.5 μg/mL) for an additional 24 h. (A) The mRNA levels of IL-1β. (B) The mRNA levels of IL-6. The release of IL-1β (C) and IL-6 (D) in LPS-stimulated RAW264.7 cells were measured using ELISA kits. Data are represented as mean ± SD, n = 3. ^#^ *p* < 0.05, ^##^ *p* < 0.01, ^###^ *p* < 0.001, ^####^ *p* < 0.0001 *vs*. the control group. * *p* < 0.05, ** *p* < 0.01, *** *p* < 0.001, **** *p* < 0.0001 *vs*. the LPS group.

To further investigate the anti-inflammatory effect of echinatin on LPS-induced RAW264.7 cells, we evaluated the expression of IL-1β and IL-6 through qRT-PCR and ELISA assay. The results showed that after LPS stimulation, the mRNA level of IL-1β was increased 3333-fold and that of IL-6 was increased 100-fold compared to the control group, whereas after echinatin (30 μM) treatment, the mRNA level of IL-1β was decreased 5.56-fold and that of IL-6 was decreased 5.26-fold compared to LPS group (Fig 3A and 3B). Furthermore, LPS treatment also resulted in an excessive release of both of these pro-inflammatory cytokines in RAW264.7 cells. However, echinatin treatment could considerably decrease the production of IL-1β and IL-6 (Fig 3C and 3D). Taken together, these results further indicated the anti-inflammatory effect of echinatin.

### Echinatin blocked LPS-induced activation of the NF-κB signaling pathway in RAW264.7 cells

**Fig 4:**
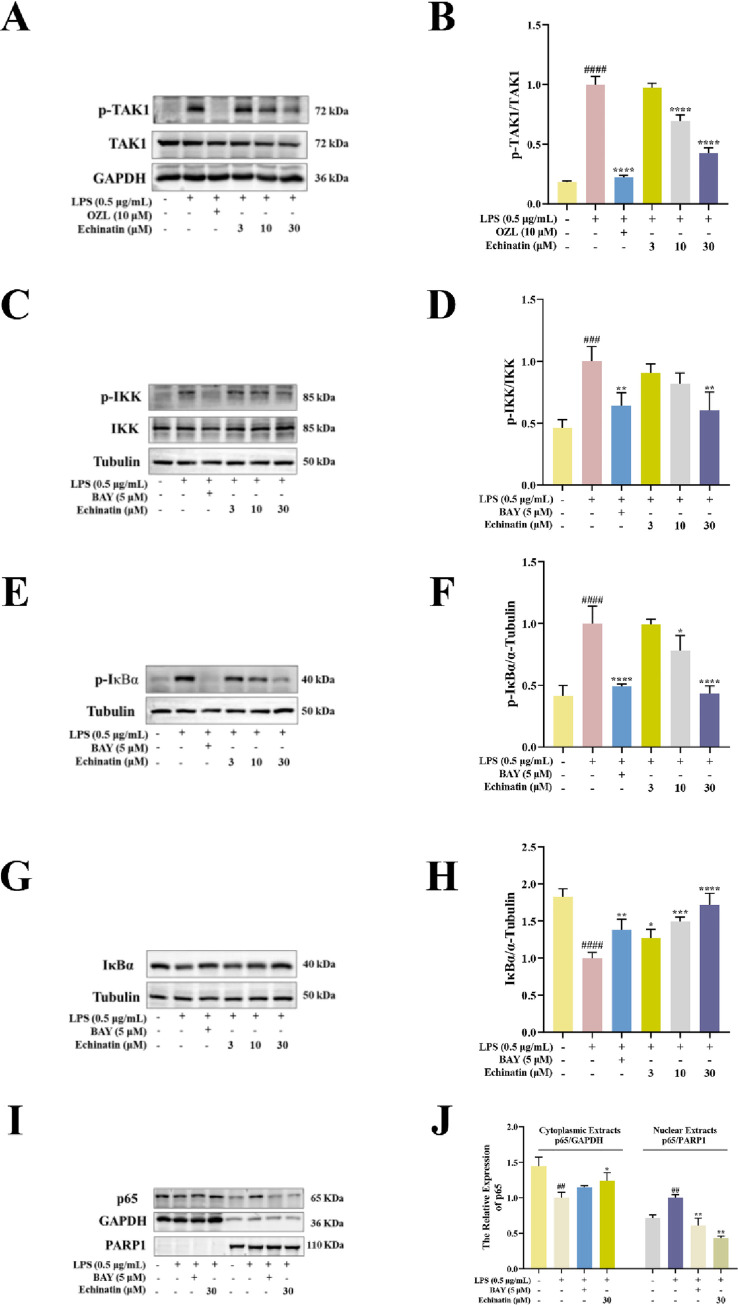
Echinatin blocks the activation of NF-κB signaling pathway in LPS-stimulated RAW264.7 cells. RAW264.7 cells were incubated with echinatin (3, 10, and 30 μM) or BAY (5 μM) for 2 h, followed by LPS (0.5 μg/mL) stimulation for an additional 5 min. The levels of p-TAK1 **(A)**, p-IKK **(C)**, and p-IκBα **(E)**, total protein levels of TAK1 **(A)**, IKK **(C)**, and IκBα **(G)**, as well as GAPDH and tubulin were detected by Western blot analysis. The quantitative analysis of the ratio of p-TAK1 **(B)**, p-IKK **(D)**, p-IκBα **(F)**, and IκBα **(H)** normalized to the loading control is indicated. RAW264.7 cells were pre-treated with echinatin or BAY for 2 h, followed by LPS-stimulating for an additional 30 min. **(I)** The cytoplasmic and nuclear fraction technology was used to measure the distribution of p65. GAPDH was used as a cytoplasmic loading control, whereas PARP1 was used as nuclear loading control. **(J)** The quantitative analysis of the ratio of p65 normalized to GAPDH or PARP1 is shown. Data are represented as mean ± SD. n = 3. ^**#**^ *p* < 0.05, ^**##**^ *p* < 0.01, ^**###**^ *p* < 0.001, ^**####**^ *p* < 0.0001 *vs*. the control group. ^*^ *p* < 0.05, ^**^ *p* < 0.01, ^***^ *p* < 0.001, ^****^ *p* < 0.0001 *vs*. the LPS group.

NF-κB signaling pathway plays a crucial role in many inflammatory diseases. To elucidate the underlying mechanism of echinatin-mediated anti-inflammatory effect, we performed a Western blot assay to determine the effect of echinatin on the activation of the NF-κB pathway. LPS stimulation markedly increased the phosphorylation of TAK1 (an upstream kinase of the NF-κB pathway), whereas echinatin pretreatment downregulated the phosphorylation of TAK1 in a dose-dependent manner, similar to 5Z-7-Oxozeaenol, a known TAK1 inhibitor (Fig 4A and 4B). Moreover, we found that echinatin or BAY (5 μM) could inhibit the phosphorylation of IKK and IκBα, as well as the degradation of IκBα in LPS-activated RAW264.7 cells (Fig 4C–4H). Furthermore, the nuclear and cytoplasmic protein extraction analysis indicated that pretreatment with echinatin or BAY could inhibit the nuclear accumulation of p65 in LPS-induced RAW264.7 cells (Fig 4I and 4J). Taken together, the above results indicated that echinatin may exert anti-inflammatory effects by inhibiting TAK1-IKK-NF-κB signaling pathway.

### Echinatin blocked LPS-induced phosphorylation of MAPK signaling pathway

**Fig 5:**
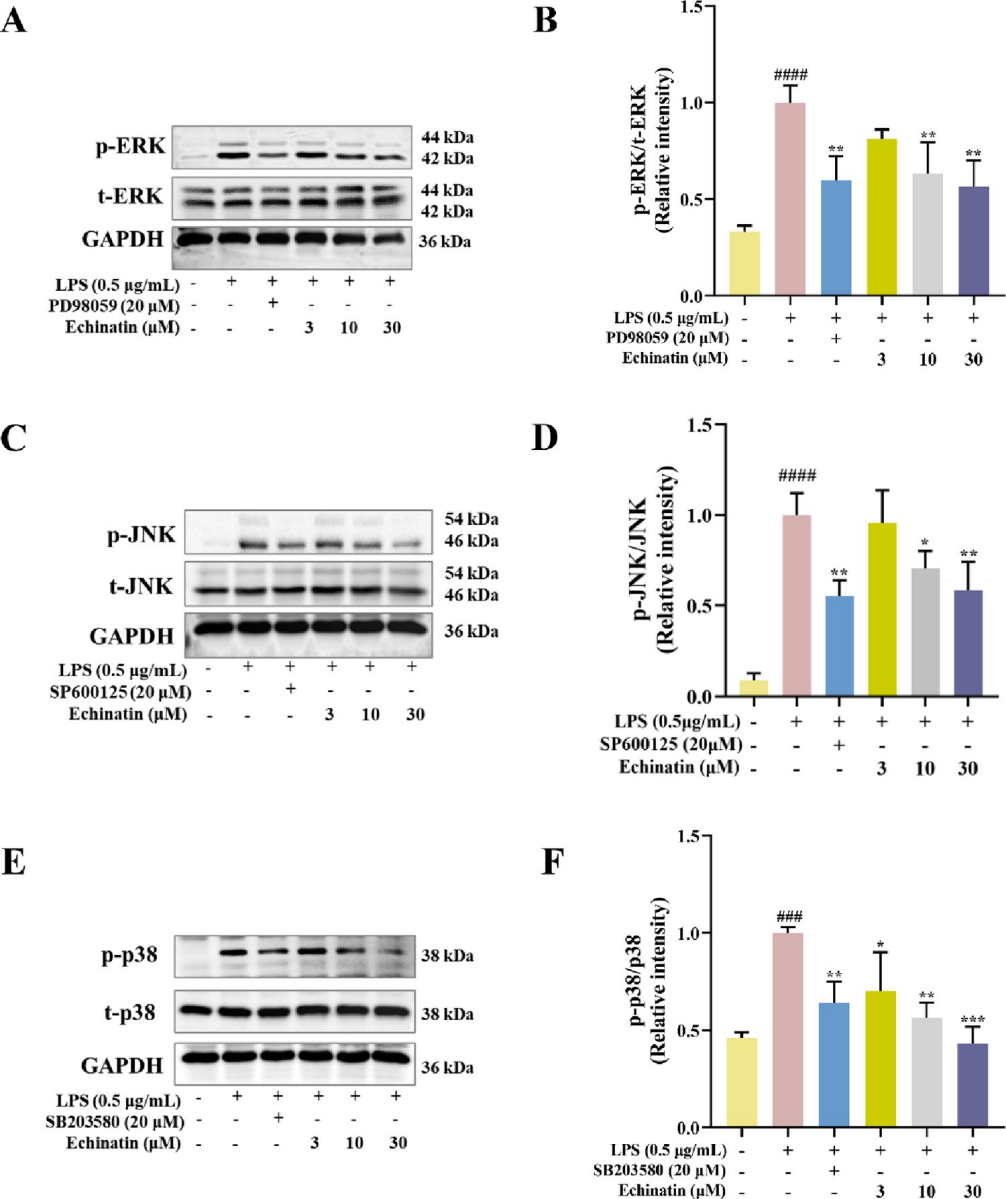
Echinatin represses the phosphorylation of MAPK in LPS-induced RAW264.7 cells. After treatment with echinatin or PD98059 (20 μM), SP600125 (20 μM) and SB203580 (20 μM) for 2 h, the cells were incubated with LPS (0.5 μg/mL) for an additional 30 min. The phosphorylated and total ERK **(A)**, JNK **(C)** and p38 **(E)** were detected by Western blotting. The statistical results of the ratio of p-ERK **(B)**, p-JNK **(D)** and p-p38 **(F)** normalized to the loading control are indicated. Data are represented as mean ± SD. n = 3. ^**#**^ *p* < 0.05, ^**##**^ *p* < 0.01, ^**###**^ *p* < 0.001, ^**####**^ *p* < 0.0001 *vs*. the control group. ^*^ *p* < 0.05, ^**^ *p* < 0.01, ^***^ *p* < 0.001, ^****^ *p* < 0.0001 *vs*. the LPS group.

MAPK signaling pathway including ERK, JNK and p38, has been shown to participate in LPS-induced inflammatory response [27]. Therefore, we detected whether MAPK pathway is involved in the anti-inflammatory effect of echinatin in LPS-stimulated RAW264.7 cells. The results showed that LPS treatment significantly increased the phosphorylation of ERK, JNK and p38. Interestingly, prior treatment with echinatin could distinctly lessen the phosphorylation levels of ERK (Fig 5A and 5B), JNK (Fig 5C and 5D), and p38 (Fig 5E and 5F) in a dose-dependent manner. Notably, PD98059 (ERK inhibitor), SP600125 (JNK inhibitor) and SB203580 (p38 inhibitor) could also suppress the LPS-induced activation of MAPK. Therefore, these results indicated that the protective effect of echinatin against LPS-induced inflammation was partially by blocking the activation of MAPK signaling pathway.

### Echinatin regulated Nrf2-HO-1 signaling pathway in RAW264.7 cells

**Fig 6:**
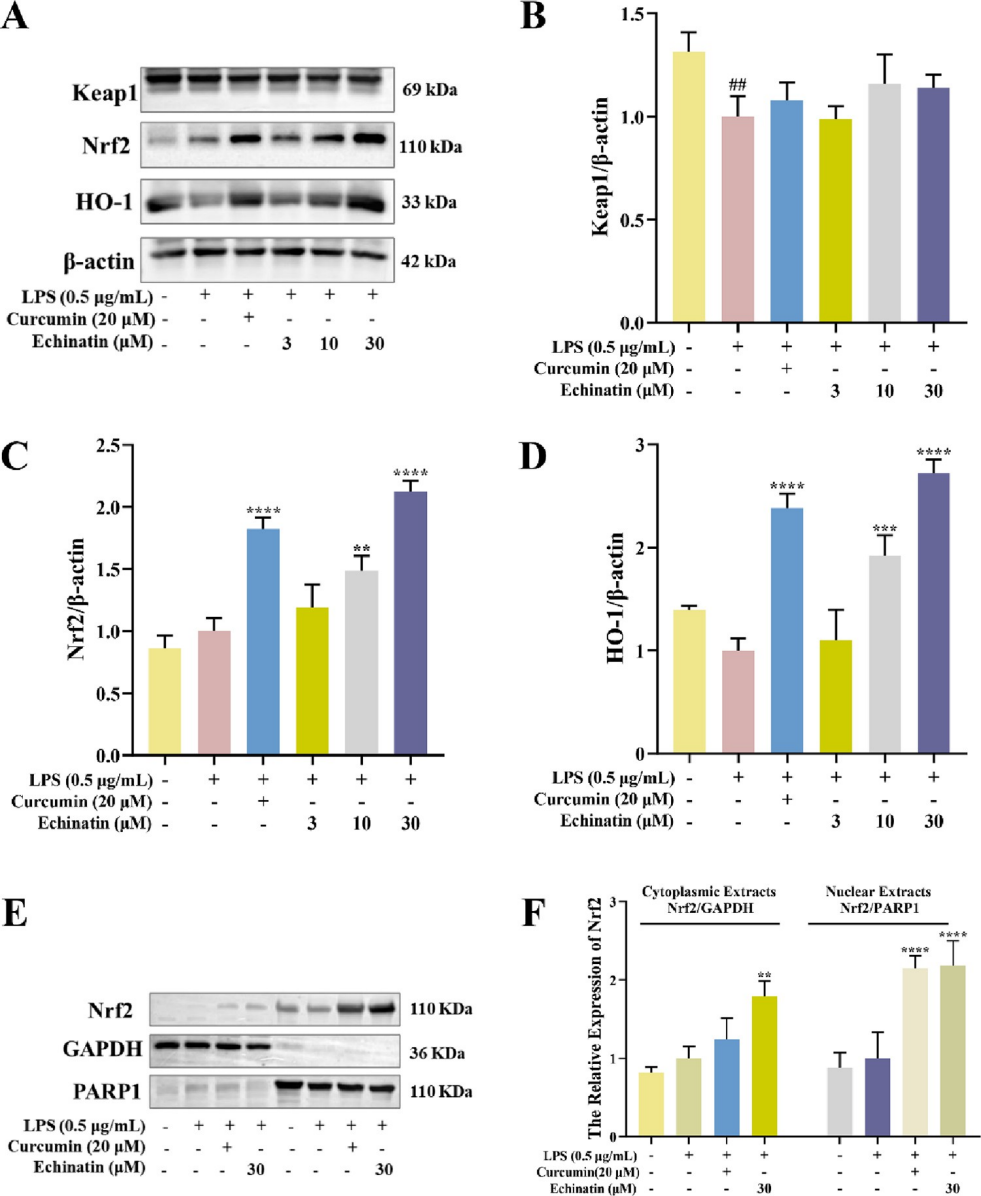
Echinatin activates Nrf2-HO-1 anti-oxidant signaling in LPS-induced RAW264.7 cells. RAW264.7 cells were treated with echinatin or curcumin for 1 h, followed by LPS (0.5 μg/mL) stimulation for an additional 8 h. **(A)** The protein levels of Keap1, Nrf2, and HO-1 were detected by Western blot assay. The quantitative results of the ratio of Keap1 **(B)**, Nrf2 **(C)**, and HO-1 **(D)** normalized to the β-actin are presented. **(E)** The cytoplasmic and nuclear fraction technology was used to measure the distribution of Nrf2. GAPDH was used as a cytoplasmic loading control, whereas PARP1 was used as a nuclear loading control. **(F)** The quantitative analysis of the ratio of Nrf2 normalized to GAPDH or PARP1 is shown. Data are represented as mean ± SD. n = 3. ^**#**^ *p* < 0.05, ^**##**^ *p* < 0.01, ^**###**^ *p* < 0.001, ^**####**^ *p* < 0.0001 *vs*. the control group. ^*^ *p* < 0.05, ^**^ *p* < 0.01, ^***^ *p* < 0.001, ^****^ *p* < 0.0001 *vs*. the LPS group.

To investigate whether Keap1-Nrf2-HO-1 signaling pathway plays an important role in the protective effect of echinatin against LPS-induced inflammatory response in RAW264.7 cells, the expression levels of Keap1, Nrf2 and HO-1 were further inspected by Western blot assay. Compared with the control group, the expression of Keap1 in RAW264.7 cells was remarkably inhibited after LPS stimulation but did not change significantly after the treatment with echinatin (Fig 6A and 6B). However, echinatin (3, 10, and 30 μM) treatment increased the expression of Nrf2 in a dose-dependent manner compared with the LPS-treated group (Fig 6C). Furthermore, the nuclear and cytoplasmic protein extraction analysis showed that echinatin markedly increased the levels of Nrf2 protein in the cytoplasmic and nuclear fractions (Fig 6E and 6F). The expression of HO-1 was considerably upregulated by echinatin treatment in LPS-incubated RAW264.7 cells (Fig 6D). The above results indicated that echinatin exerted antioxidative effects by activating the Nrf2-HO-1 signaling pathway, thereby exhibiting a potent anti-inflammatory activity.

### Echinatin interacts with TAK1 and Keap1

**Fig 7:**
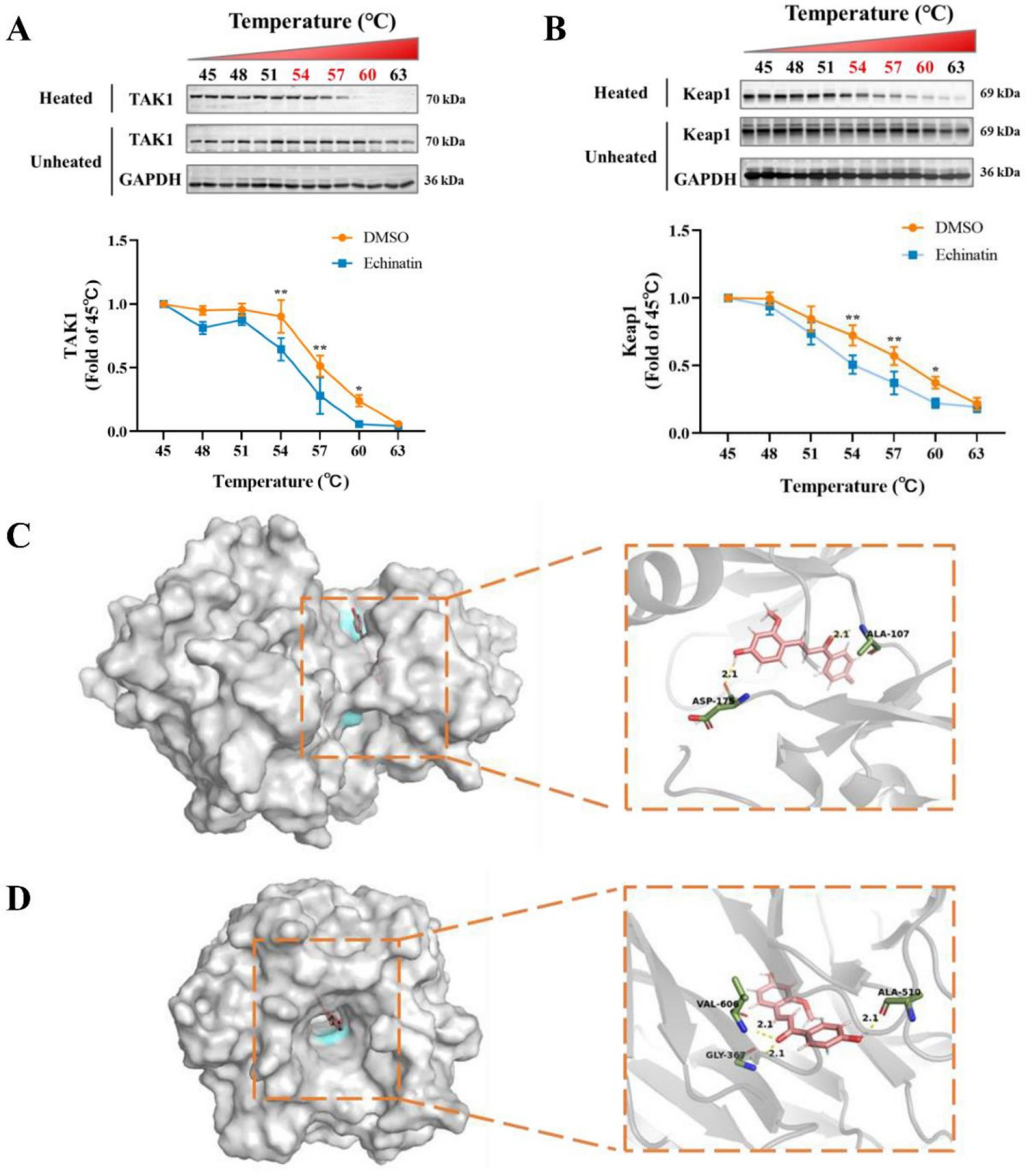
Echinatin directly interacts with TAK1 and Keap1. The effects of echinatin on the thermal stability of TAK1 **(A)** and Keap1 **(B)** were detected by CETSA and Western blotting. The top panel shows the quantification of the thermal stability of TAK1 and Keap1 in the range of 45°C to 63°C. The optical densities of TAK1 and Keap1 were normalized to those obtained at 45°C. **(C)** The 3D representation of echinatin binding to TAK1 (PDB: 4O91) protein. **(D)** The 3D representation of the interaction of echinatin at the active site of Keap1 (PDB: 6LRZ). Data are represented as mean ± SD. n = 3. ^*^ *p* < 0.05, ^**^ *p* < 0.01, ^***^ *p* < 0.001, ^****^ *p* < 0.0001.

The above results showed that echinatin might improve inflammation in LPS-induced RAW264.7 cells by inhibiting the NF-κB and MAPK signaling pathways and activating the Nrf2 pathway. Because TAK1 is a vital upstream kinase, mediating the activation of MAPK and NF-κB pathways [28]. Therefore, we further investigated whether echinatin directly interacts with TAK1, resulting in the inhibition of MAPK and NF-κB pathways. As shown in Fig 7A, compared with the DMSO group, echinatin (100 μM) markedly decreased the thermal stability of TAK1 at 54–60°C in RAW264.7 cell lysates. This result indicated a direct interaction between echinatin and TAK1. However, echinatin did not alter the thermal stability of IκBα (S1A Fig in S1 File), another important protein that participates in the activation of NF-κB. In addition, we adopted a molecular docking technique to determine the binding between TAK1 and echinatin. As depicted in Fig 7C and Table 1, echinatin could form hydrogen bonds with TAK1 via Ala-107 and Asp-175. Taken together, the above results indicated that echinatin might block the activation of the MAPK and NF-κB signaling pathways by directly interacting with TAK1, thereby inhibiting the inflammatory response.

**Table 1:** Molecular docking between echinatin with Keap1 and TAK1.

Ligand	Compound CID	Protein	PDB ID	Docking score (kcal/mol)	Protein-ligand interaction (H-bond)
**Echinatin**	6442675	Keap1	6LRZ	-6.432	Gly-367, Ala-510 and Val-606
		TAK1	4O91	-8.704	Ala-107 and Asp-175

Furthermore, the previous study showed that Keap1 acts as a negative regulator of the Nrf2 pathway [29]. Therefore, we investigated whether echinatin has a direct interaction with Keap1, leading to the activation of the Nrf2-HO-1 signaling pathway. CETSA result suggested that echinatin remarkably decreased the thermal stability of Keap1 at 54–60°C in RAW264.7 cell lysates (Fig 7B). However, echinatin did not alter the thermal stability of Nrf2 protein (S1B Fig in S1 File). Moreover, the molecular docking assay showed that echinatin formed hydrophobic bonds with several residues of Keap1, including Gly-367, Ala-510, and Val-606 (Fig 7D and Table 1). Therefore, these results indicated that echinatin might potentially interact with Keap1 proteins, and further activate the Nrf2-HO-1 pathway.

### Echinatin ameliorated LPS-induced ALI *in vivo*

**Fig 8:**
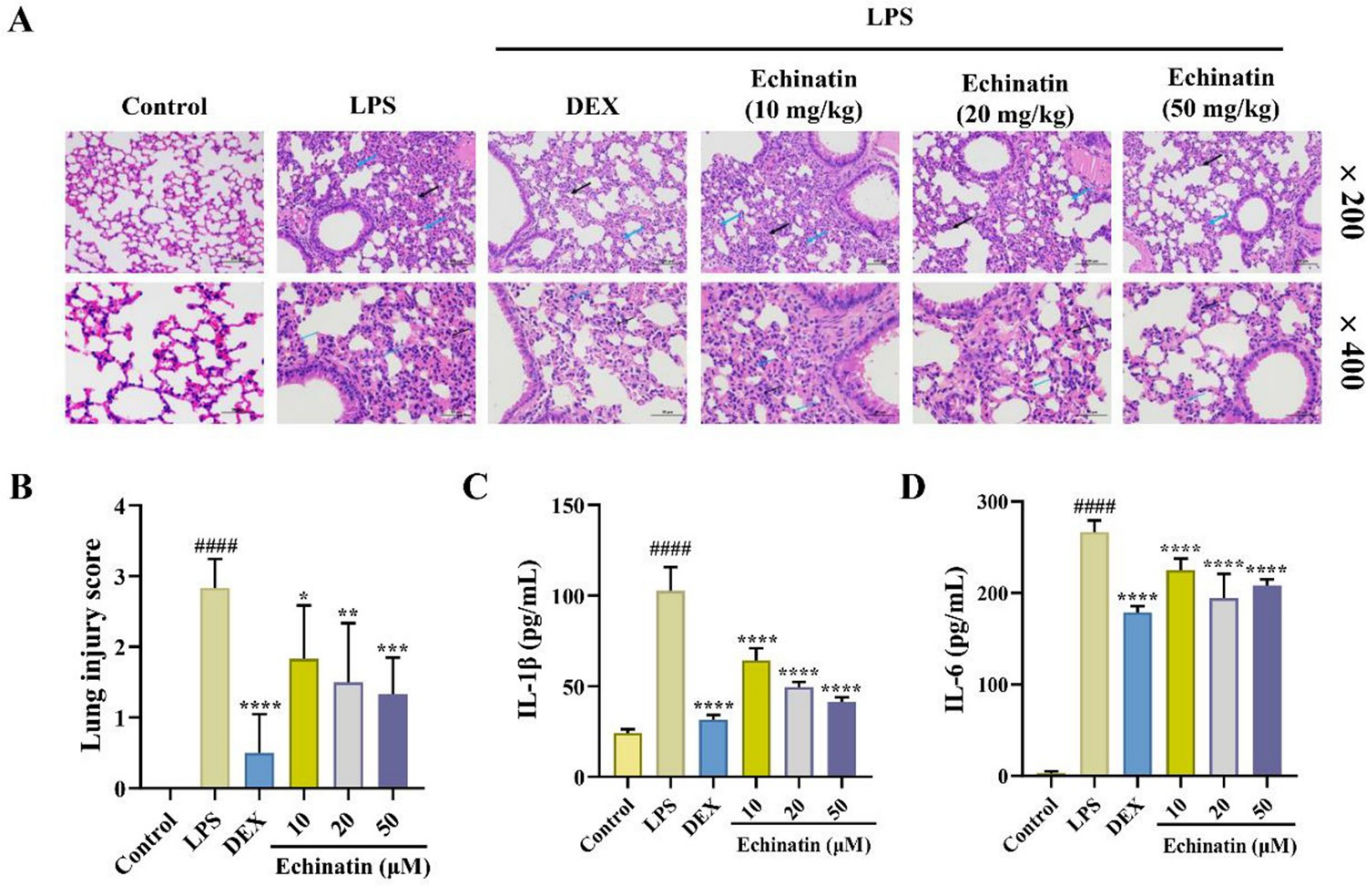
Echinatin ameliorates LPS-induced ALI in mice. Mice were administrated with echinatin (10, 20, and 50 mg/kg) or DEX (0.1 mg/kg), or vehicle for 2 h, followed by the tail vein injection of LPS (10 mg/kg) for another 12 h. **(A)** H&E staining was used to access pathological changes observed in lung tissues. Scale bar = 100 μm (×200) and 50 μm (×400). The black arrow replaces alveolar septa widening and the blue arrow replaces neutrophil infiltration. **(B)** Score of the lung injury. Effects of echinatin on the serum levels of IL-1β **(C)** and IL-6 **(D)** in LPS-stimulated ALI mice were analyzed by ELISA. Data are represented as mean ± SD, n = 6. ^**#**^ *p* < 0.05, ^**##**^ *p* < 0.01, ^**###**^ *p* < 0.001, ^**####**^ *p* < 0.0001 *vs*. the control group. ^*^ *p* < 0.05, ^**^ *p* < 0.01, ^***^ *p* < 0.001, ^****^ *p* < 0.0001 *vs*. the LPS group.

*In vivo*, we evaluated the protective effect of echinatin on LPS-induced ALI. As shown in Fig 8A, the tail intravenous injection of LPS in mice caused ALI. Compared with control group, the significant pathological changes in pulmonary tissues included alveolar wall thickening, alveolar atrophy, alveolar septa widening, and neutrophil infiltration. Interestingly, echinatin or DEX could distinctly ameliorate LPS-induced pulmonary inflammatory injury, especially neutrophil infiltration. Furthermore, the results of pathological examination demonstrated that both echinatin and DEX could significantly reduce the increase of LPS-induced lung injury scores in mice (Fig 8B). Furthermore, several of the cytokines are also involved in the progression of ALI, including IL-1β and IL-6 [3]. Consistent with the histological examination staining results, tail intravenous LPS injection markedly increased the serum levels of IL-1β and IL-6, and echinatin or DEX could reverse the production of these pro-inflammatory cytokines (Fig 8C and 8D). Taken together, these results indicated that echinatin could effectively ameliorate LPS-induced ALI *in vivo*.

## Discussion

Currently, the drug treatment of ALI has limited efficacy and distinct toxic and side effects, thus, there is an urgency to explore new therapeutic medicine. In this study, we discovered that echinatin exerted anti-inflammatory effects on LPS-stimulated macrophages and LPS-stimulated ALI mice by regulating the TAK1-MAPK/NF-κB and Nrf2-HO-1 signaling pathways. Therefore, this study provides a new perspective for explaining the anti-inflammatory mechanism of echinatin.

Generally, the most common cause of ALI is a bacterial infection, including Gram-negative bacteria [4]. The exposure of LPS, the most critical component of the cell membrane of Gram-negative bacteria, to the lung tissues can trigger inflammatory cell infiltration, thus resulting in inflammatory responses and oxidative stress [5, 30]. Therefore, LPS-induced ALI in mice is an effective model for studying the pathogenesis of ALI and evaluating anti-inflammatory drugs. In this study, we found that the tail intravenous injection of LPS in mice caused a distinct pulmonary inflammatory response, especially significant neutrophil infiltration. Conversely, echinatin pretreatment markedly alleviated pulmonary inflammatory damage in LPS-stimulated ALI mice. Furthermore, the excessive production of various pro-inflammatory cytokines and mediators can accelerate ALI progression [31]. The immune system is activated during a bacterial invasion, which releases various pro-inflammatory cytokines and initiates lung injury [32]. Our study showed that echinatin intervention distinctly decreased the serum levels of IL-1β and IL-6 in the ALI mice, thereby exhibiting anti-inflammatory activity *in vivo*. Moreover, Xu *et al*. [22] reported that echinatin could ameliorate LPS-induced septic shock in mice and decrease the release of TNF-α and IL-1β, which further supported our conclusion that echinatin exhibited a tremendous anti-inflammatory activity *in vivo*.

Macrophages are immune sentinels that play an essential role in inflammatory response amplification and regression, mainly through the production of a range of pro-inflammatory cellular mediators, inducible synthases and pro-inflammatory cytokines, such as NO, PGE_2_, iNOS, COX-2, IL-1β and IL-6 [33, 34]. In the present study, we found that echinatin could inhibit LPS-induced increase in iNOS and COX-2 protein levels in MH-S murine alveolar macrophages, thereby inhibiting NO and PGE_2_ production to exert an anti-inflammatory effect, and similar results were observed in RAW264.7 macrophages. LPS, a common cause of ALI, binds to the TLR4/MD2 complex and initiates the recruitment of the intracellular molecular adaptor MyD88, followed by the activation of multiple signaling pathways, such as NF-κB and MAPK signaling pathways [11]. The NF-κB pathway is closely associated with ALI pathogenesis, and its inhibition can significantly improve lung injury in ALI mice [35, 36]. Therefore, blocking NF-κB pathway activation might be a prospective therapeutic strategy against ALI. Normally, NF-κB interacts with IκB, and its inactive dimer is present in the cytoplasm. However, some pro-inflammatory stimulants, such as LPS, can activate IKK, which further phosphorylates IκBα, thereby degrading IκBα in a ubiquitination-dependent manner, releasing the NF-κB complex [37]. Moreover, activated NF-κB can translocate from the cytoplasm to the nuclear and drive transcription and translation of various target genes [38]. The present results indicated that pretreatment with echinatin suppressed the LPS-induced increase in IKK and IκBα phosphorylation levels, as well as IκBα degradation and p65 nuclear translocation in the RAW264.7 cells. These results may well explain the anti-inflammatory mechanism of echinatin in relation to the NF-κB pathway inhibition. MAPK signaling is a classic pathway in various inflammatory diseases such as ALI, which is characterized by the phosphorylation of ERK, JNK and p38 [39]. Previous studies have shown that the pharmacological blockade of MAPK markedly reduces LPS-induced inflammatory responses and ameliorates lung pathology deterioration in LPS-stimulated ALI mice [31, 40]. Similarly, the present findings suggested that pretreatment with echinatin inhibited the phosphorylation of ERK, JNK and p38, which contributing to relieve the LPS-induced inflammatory response in RAW264.7 cells.

TAK1 is a pivotal upstream signaling component of the NF-κB and MAPK signaling pathways [41]. Studies have shown that some drugs act as anti-inflammatory agents by inhibiting TAK1, indicating that TAK1 inhibition is an effective therapeutic strategy against ALI [42, 43]. CETSA, a valuable method based on the ligand binding-induced thermal transfer of the target proteins, verifies interactions between drugs and protein targets and examines the thermal stability of proteins induced by drugs [42, 44]. In this study, the CETSA results suggested that echinatin distinctly decreased the thermal stability of TAK1 at 54–60°C, suggesting that echinatin directly interacted with TAK1. Furthermore, the Western blotting results showed that echinatin pretreatment repressed TAK1 phosphorylation in a dose-dependent manner, further supporting the CETSA results. Moreover, the molecular docking results showed that echinatin formed two hydrogen bonds with TAK1 at Ala-107 and Asp-175, suggesting the high binding potential of echinatin to TAK1. Previous studies have suggested that drugs can suppress TAK1 activity by forming a hydrogen bond with Ala-107 in TAK1, leading to an inhibitory effect on the NF-κB signaling pathway [33, 45]. Thus, we speculated that echinatin might bind directly to TAK1, thereby blocking MAPK/NF-κB signaling pathway activation and demonstrating that TAK1 inhibition is a valuable strategy for treating inflammation-mediated diseases.

Accumulating evidences had reported that oxidative stress is critical in inflammation-mediated diseases, such as ALI and sepsis [40]. Nrf2 participates in the regulation of multiple cellular responses, including oxidative stress, and is a redox-sensitive transcription factor [46]. Keap1, the main repressor of Nrf2, binds to Nrf2 and accelerates Nrf2 ubiquitinated degradation under normal conditions [47, 48]. However, under oxidative and electrophilic stress conditions, Nrf2 separates from the Keap1-Nrf2 complex [49]. Then, this activated Nrf2 undergoes nuclear translocation into the nucleus and combines with ARE, to up-regulate gene expression of HO-1 and exert antioxidant effects, thereby suppressing the NF-κB pathway and inflammatory response [31, 50]. Therefore, activating the Nrf2/HO-1 signaling pathway is a feasible option for intervention in inflammatory diseases. The present study showed that echinatin markedly reduced the thermal stability of Keap1 at 54–60°C according to the CETSA method, indicating that echinatin directly interacted with Keap1. Subsequently, the Western blot analysis results showed that echinatin had no significant effect on Keap1 but increased Nrf2 levels in LPS-stimulated RAW264.7 cells. The results of molecular docking suggested that echinatin might occupy pivotal residues required for the binding of Keap1 to Nrf2, such as Gly-367, Ala-510, and Val-606. Previous studies have reported that several compounds exhibited potent anti-oxidant effects via interacting with Gly-367 and Val-606 of Keap1 [51, 52]. Combining with the present results, we presumed that echinatin might occupy the critical binding pocket of Keap1, blocking the interaction between Keap1 and Nrf2, thereby increasing Nrf2 nuclear translocation and exerting anti-oxidative effects. Thus, the above results demonstrated that echinatin could inhibit LPS-stimulated inflammatory response by regulating the Nrf2-HO-1 pathway.

## Conclusion

**Fig 9:**
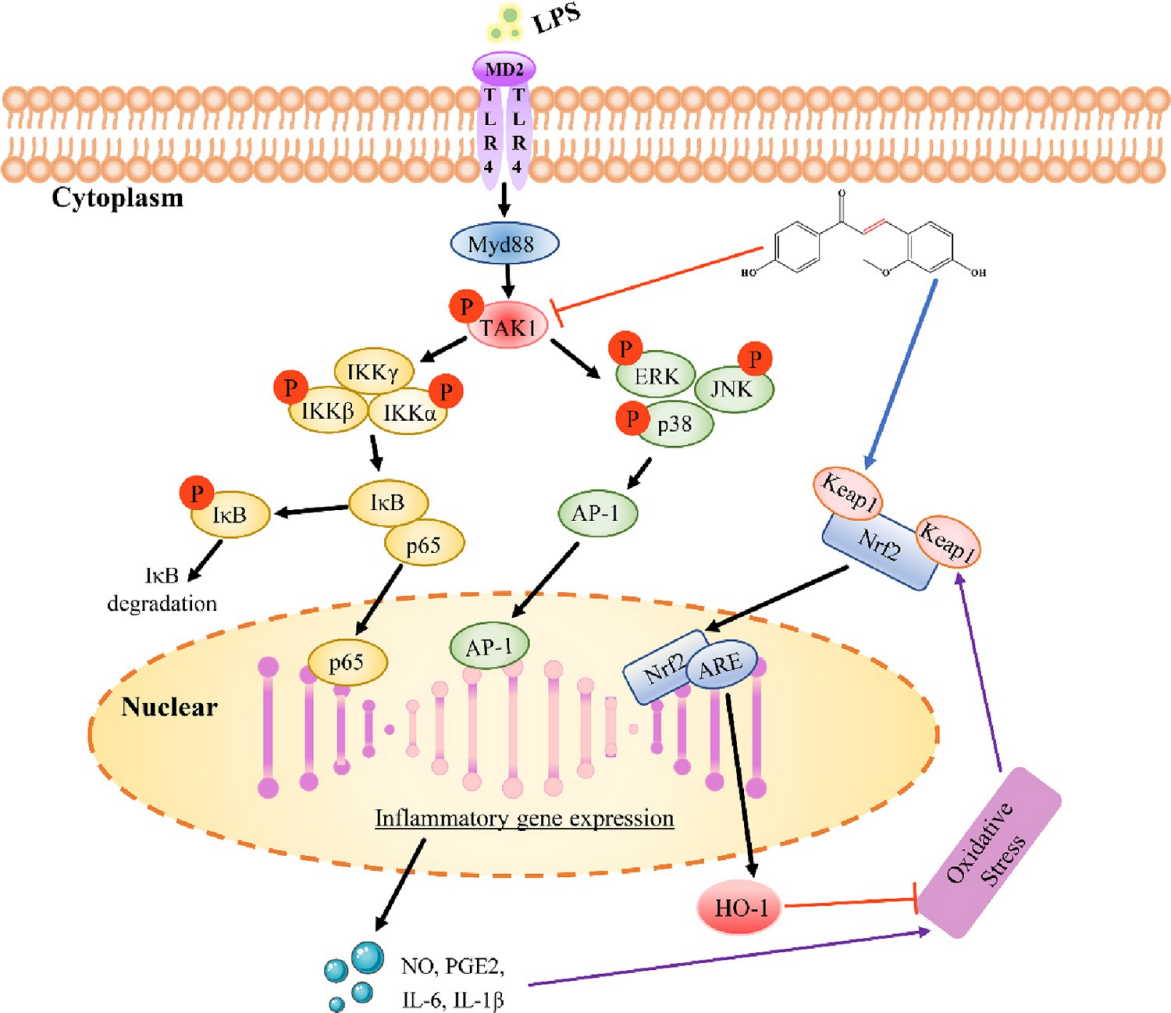
The molecular mechanism of action of echinatin exerting anti-inflammatory effects in macrophages.

To conclude, the present study showed that echinatin could attenuate inflammatory responses induced by LPS in macrophages and ALI mice, which might be related to inhibiting the TAK1-MAPK/NF-κB signaling pathway and activating the Nrf2-HO-1 signaling pathway (Fig 9). Therefore, this study demonstrated that echinatin could be a candidate for the treatment of ALI. Moreover, the findings also contributed to explaining the application of licorice to inflammatory diseases.

## Supporting information

S1 Table The primers of target genes. (DOCX)

S1 File (ZIP)

S1 Raw images (PDF)

## Abbreviations

ALI: acute lung injury
ARDS: acute respiratory distress syndrome
CCK-8: cell counting kit-8
CETSA: cellular thermal shift assay
COX-2: cyclooxygenase 2
Cys: cysteine
DMEM: Dulbecco’s Modified Eagle’s Medium
DEX: dexamethasone
DMSO: dimethyl sulfoxide
ELISA: enzyme linked immunosorbent assay
ERK: extracellular regulated protein kinases
FBS: fetal bovine serum
HE: hematoxylin and eosin
HO-1: heme oxygenase-1
IκBα: inhibitor of nuclear factor-kappa B alpha
IKK: inhibitor of nuclear factor kappa B kinase
IL-1β: interleukin-1 beta
iNOS: inducible nitric oxide synthase
JNK: c-Jun N-terminal kinases
Keap1: kelch-like ECH-associated protein 1
LPS: lipopolysaccharide
MAPKs: mitogen-activated protein kinases
NF-κB: nuclear-factor kappa-B
NLRP3: NOD-like receptor thermal protein domain associated protein 3
NO: nitric oxide
Nrf2: nuclear factor erythroid 2-related factor 2
PAMPs: pathogen-associated molecular patterns
PARP1: poly [ADP-ribose] polymerase 1
PGE_2_: prostaglandin E2
PRR: pattern recognition receptor
TAK1: transforming growth factor β activated kinase-1
TLR4: toll-like receptor 4
