# Supplementary material for: Echinatin attenuates acute lung injury and inflammatory responses via TAK1-MAPK/NF-κB and Keap1-Nrf2-HO-1 signaling pathways in macrophages
Source: PLoS One. 2024 May 16;19(5):e0303556. doi: 10.1371/journal.pone.0303556 (PMC11098428; doi:10.1371/journal.pone.0303556)
Supplement: S1 Table — (DOCX) [file pone.0303556.s001.docx]

| **Genes** | **Forward primers (5′-3′)** | **Reverse primers (5′-3′)** |
| --- | --- | --- |
| **iNOS** | CCTGTGAGACCTTTGATG | CCTATATTGCTGTGGCTC |
| **COX-2** | CAACACCTGAGCGGTTAC | GTTCCAGGAGGATGGAGT |
| **IL-1β** | CAACCAACAAGTGATATTCTCCATG | GATCCACACTCTCCAGCTGCA |
| **IL-6** | TAGTCCTTCCTACCCCAATTTCC | TTGGTCCTTAGCCACTCCTTC |
| **GAPDH** | TGCACCACCAACTGCTTAGC | GGCATGGACTGTGGTCATGAG |
