# Supplementary material for: Echinatin attenuates acute lung injury and inflammatory responses via TAK1-MAPK/NF-κB and Keap1-Nrf2-HO-1 signaling pathways in macrophages
Source: PLoS One. 2024 May 16;19(5):e0303556. doi: 10.1371/journal.pone.0303556 (PMC11098428; doi:10.1371/journal.pone.0303556)
Supplement: S1 File — (ZIP) [file pone.0303556.s002.zip › Supporting Information/S1_raw gel images.pdf]

The following sections were the original images of western blot. The bottom of the band shows the name of the stripes.

Fig 1

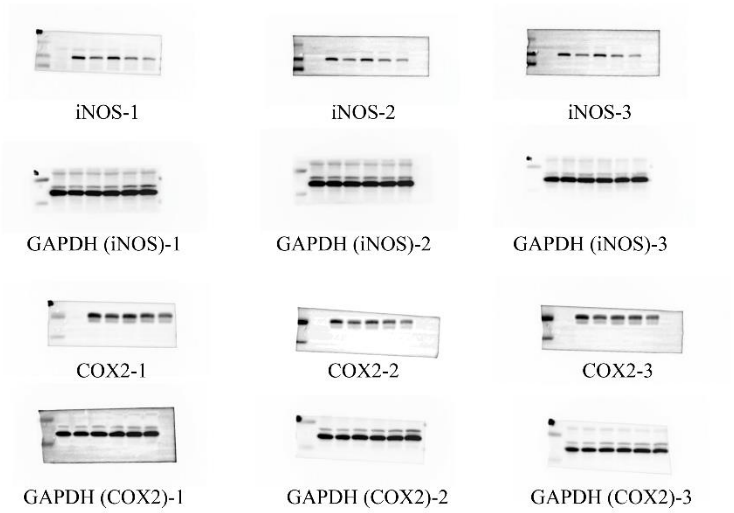

Fig 2

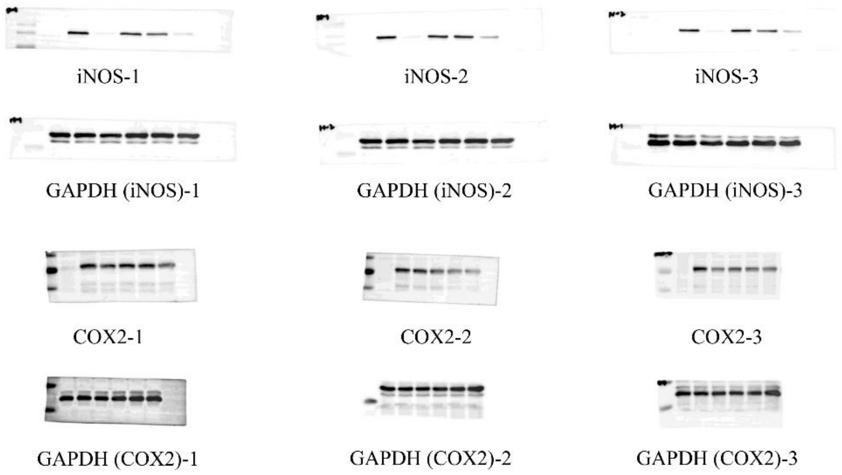

Fig 4

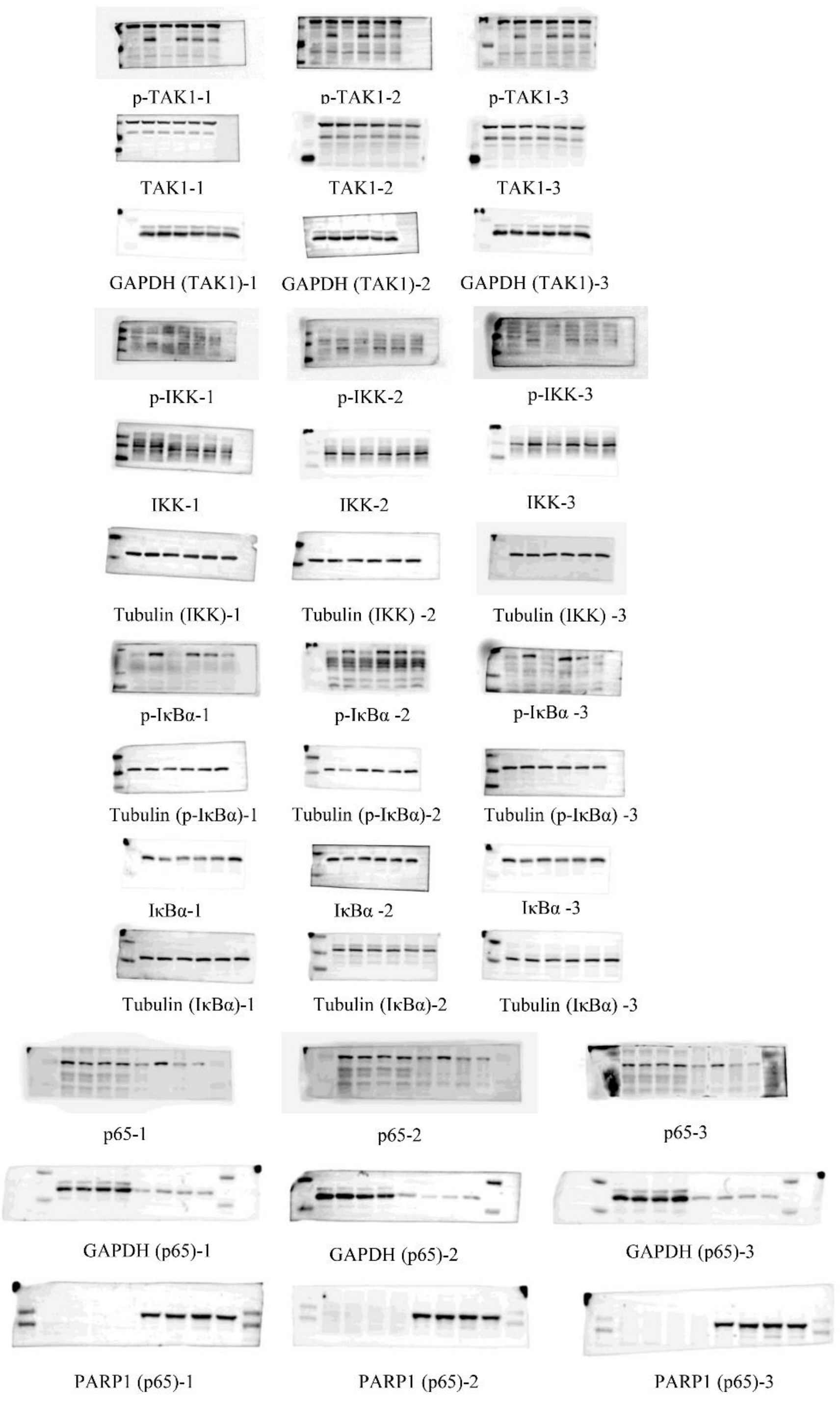

p-TAK1-1
p-TAK1-2
p-TAK1-3
TAK1-1
TAK1-2
TAK1-3
GAPDH (TAK1)-1
GAPDH (TAK1)-2
GAPDH (TAK1)-3
p-IKK-1
p-IKK-2
p-IKK-3
IKK-1
IKK-2
IKK-3
Tubulin (IKK)-1
Tubulin (IKK) -2
Tubulin (IKK) -3
p-IκBα-1
p-IκBα -2
p-IκBα -3
Tubulin (p-IκBα)-1
Tubulin (p-IκBα)-2
Tubulin (p-IκBα) -3
IκBα-1
IκBα -2
IκBα -3
Tubulin (IκBα)-1
Tubulin (IκBα)-2
Tubulin (IκBα) -3
p65-1
p65-2
p65-3
GAPDH (p65)-1
GAPDH (p65)-2
GAPDH (p65)-3
PARP1 (p65)-1
PARP1 (p65)-2
PARP1 (p65)-3

Fig 5

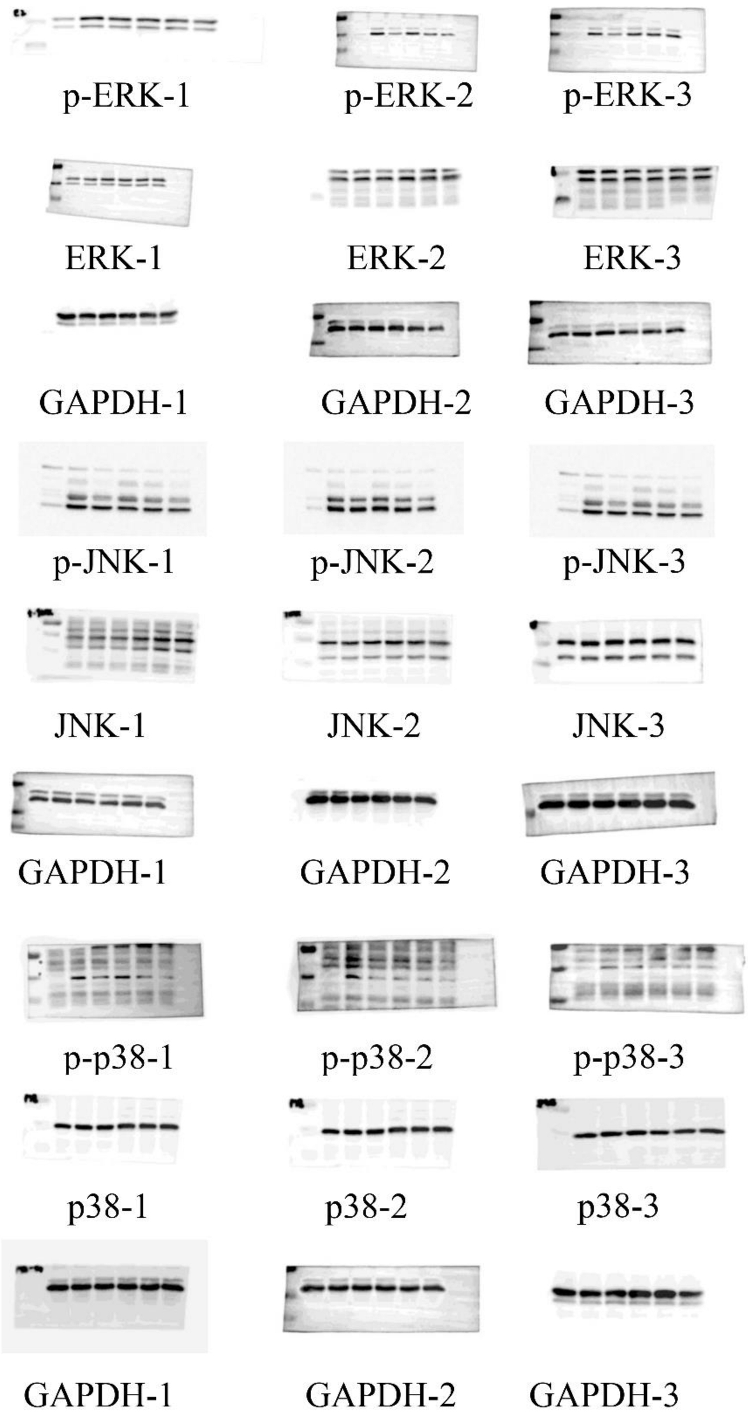
p-ERK-1
p-ERK-2
p-ERK-3
ERK-1
ERK-2
ERK-3
GAPDH-1
GAPDH-2
GAPDH-3
p-JNK-1
p-JNK-2
p-JNK-3
JNK-1
JNK-2
JNK-3
GAPDH-1
GAPDH-2
GAPDH-3
p-p38-1
p-p38-2
p-p38-3
p38-1
p38-2
p38-3
GAPDH-1
GAPDH-2
GAPDH-3

Fig 6

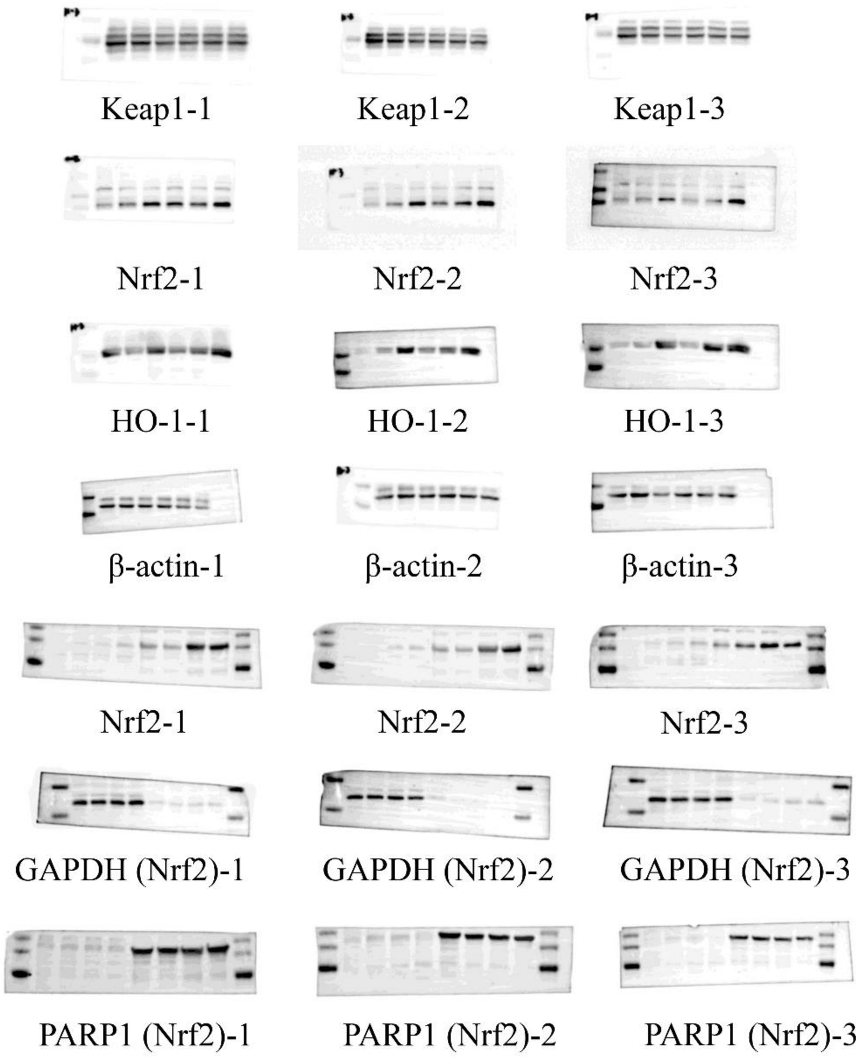
Keap1-1
Keap1-2
Keap1-3
Nrf2-1
Nrf2-2
Nrf2-3
HO-1-1
HO-1-2
HO-1-3
β-actin-1
β-actin-2
β-actin-3
Nrf2-1
Nrf2-2
Nrf2-3
GAPDH (Nrf2)-1
GAPDH (Nrf2)-2
GAPDH (Nrf2)-3
PARP1 (Nrf2)-1
PARP1 (Nrf2)-2
PARP1 (Nrf2)-3

Fig 7

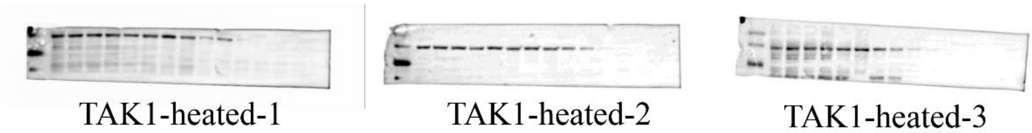

TAK1-heated-1 TAK1-heated-2 TAK1-heated-3

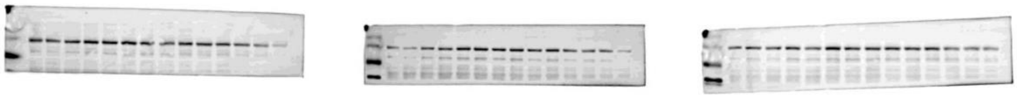

TAK1-unheated-1 TAK1-unheated-2 TAK1-unheated-3

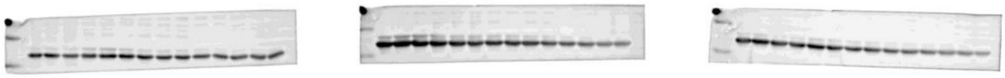

GAPDH-unheated-1 GAPDH-unheated-2 GAPDH-unheated-3

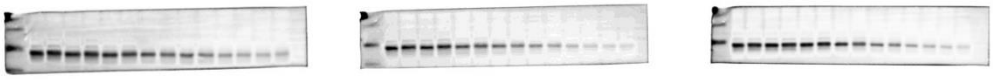

Keap1-heated-1 Keap1-heated-2 Keap1-heated-3

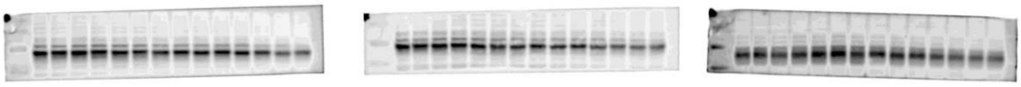

Keap1-unheated-1 Keap1-unheated-2 Keap1-unheated-3

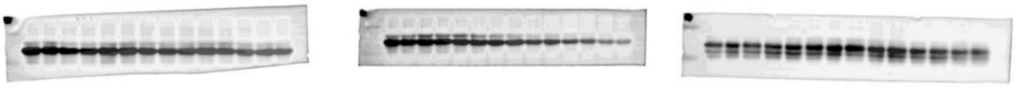

GAPDH-unheated-1 GAPDH-unheated-2 GAPDH-unheated-3

S1 Fig

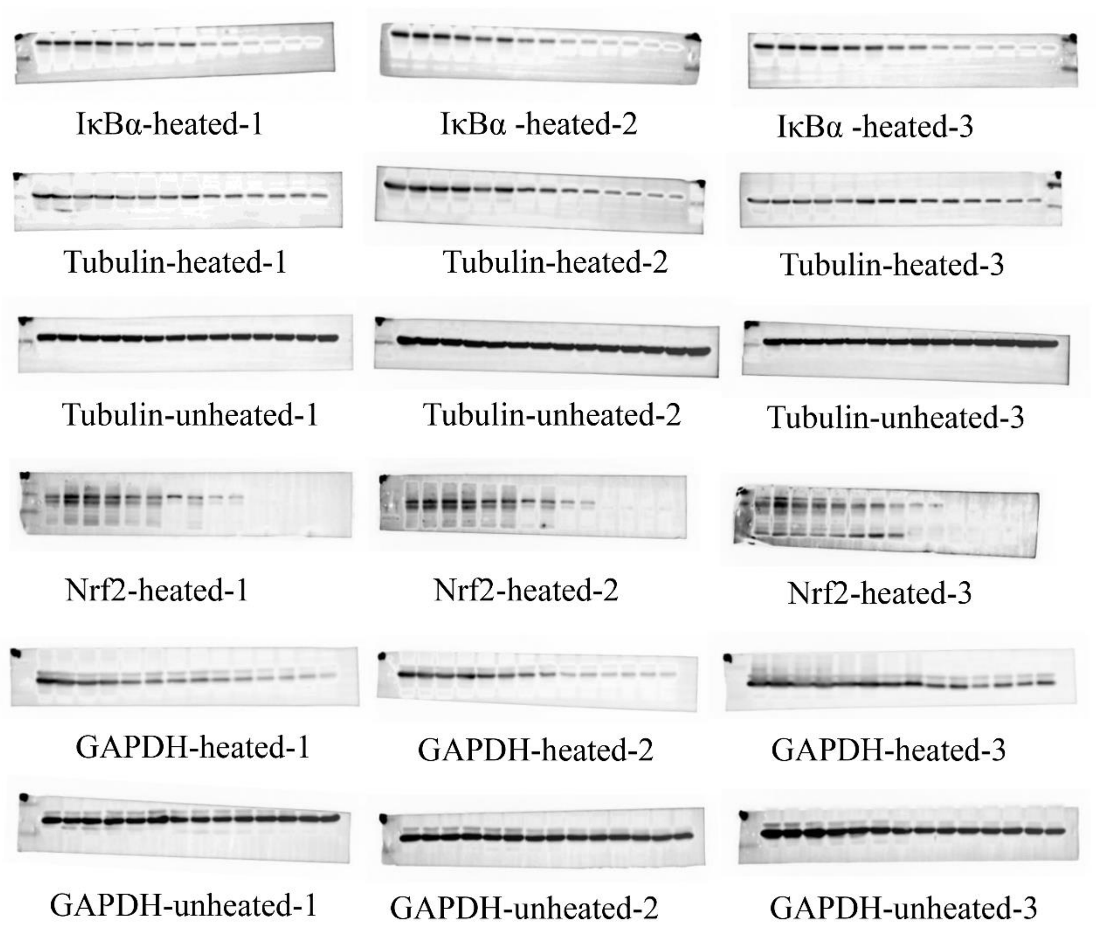
IκBα-heated-1
IκBα -heated-2
IκBα -heated-3
Tubulin-heated-1
Tubulin-heated-2
Tubulin-heated-3
Tubulin-unheated-1
Tubulin-unheated-2
Tubulin-unheated-3
Nrf2-heated-1
Nrf2-heated-2
Nrf2-heated-3
GAPDH-heated-1
GAPDH-heated-2
GAPDH-heated-3
GAPDH-unheated-1
GAPDH-unheated-2
GAPDH-unheated-3
