# Supplementary figures and images for: Echinatin attenuates acute lung injury and inflammatory responses via TAK1-MAPK/NF-κB and Keap1-Nrf2-HO-1 signaling pathways in macrophages
Source: PLoS One. 2024 May 16;19(5):e0303556. doi: 10.1371/journal.pone.0303556 (PMC11098428; doi:10.1371/journal.pone.0303556)

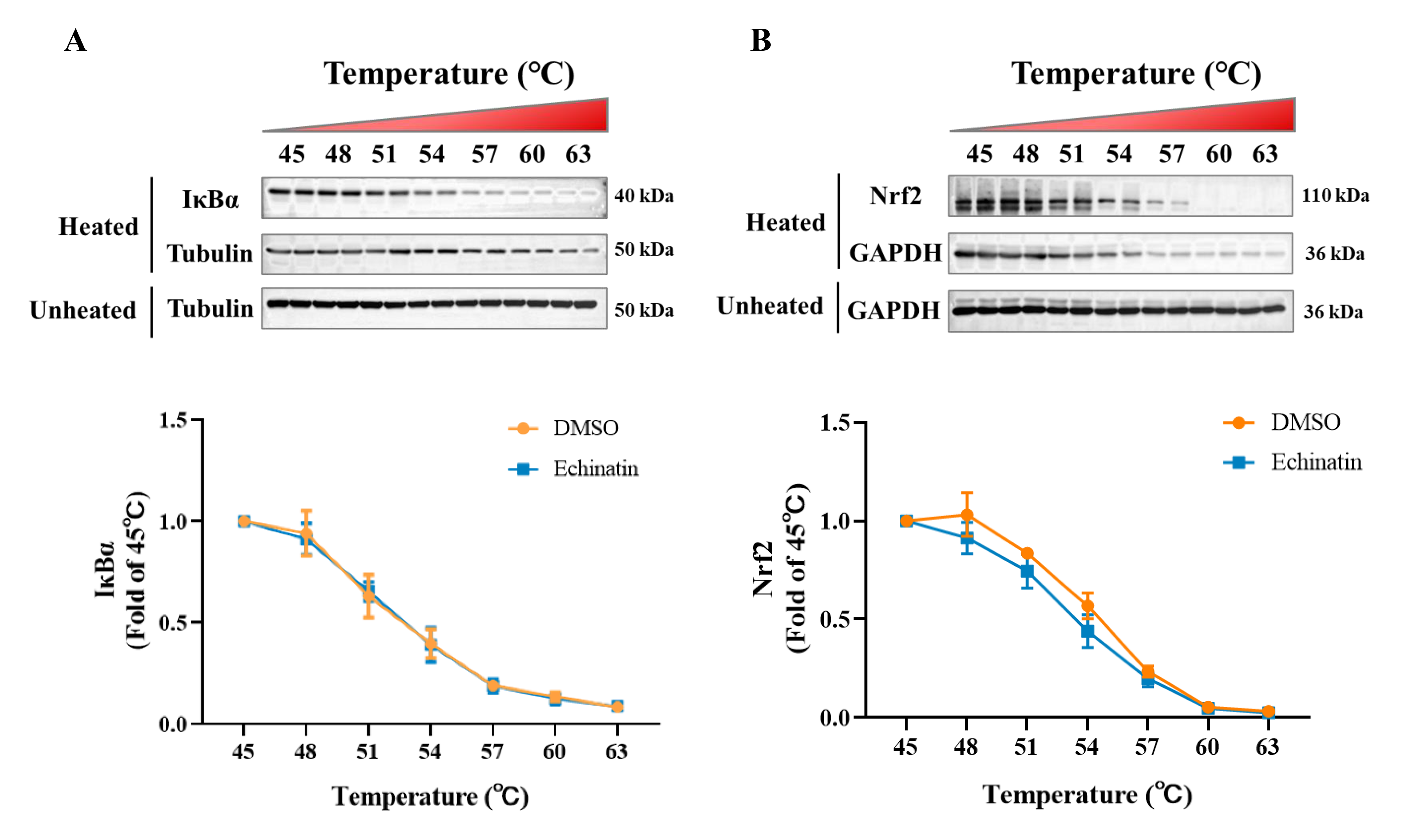

Supplement: S1 File — (ZIP) [file pone.0303556.s002.zip › Supporting Information/S1_Fig.tif]
