# Supplementary material for: Echinatin attenuates acute lung injury and inflammatory responses via TAK1-MAPK/NF-κB and Keap1-Nrf2-HO-1 signaling pathways in macrophages
Source: PLoS One. 2024 May 16;19(5):e0303556. doi: 10.1371/journal.pone.0303556 (PMC11098428; doi:10.1371/journal.pone.0303556)
Supplement: S1 Raw images — (PDF) [file pone.0303556.s003.pdf]

The following sections were the original images of Western blot. The left or right column in the figure represented the molecular weight of the protein marker, and the bottom of the band shows the name of the stripes.

Fig 1

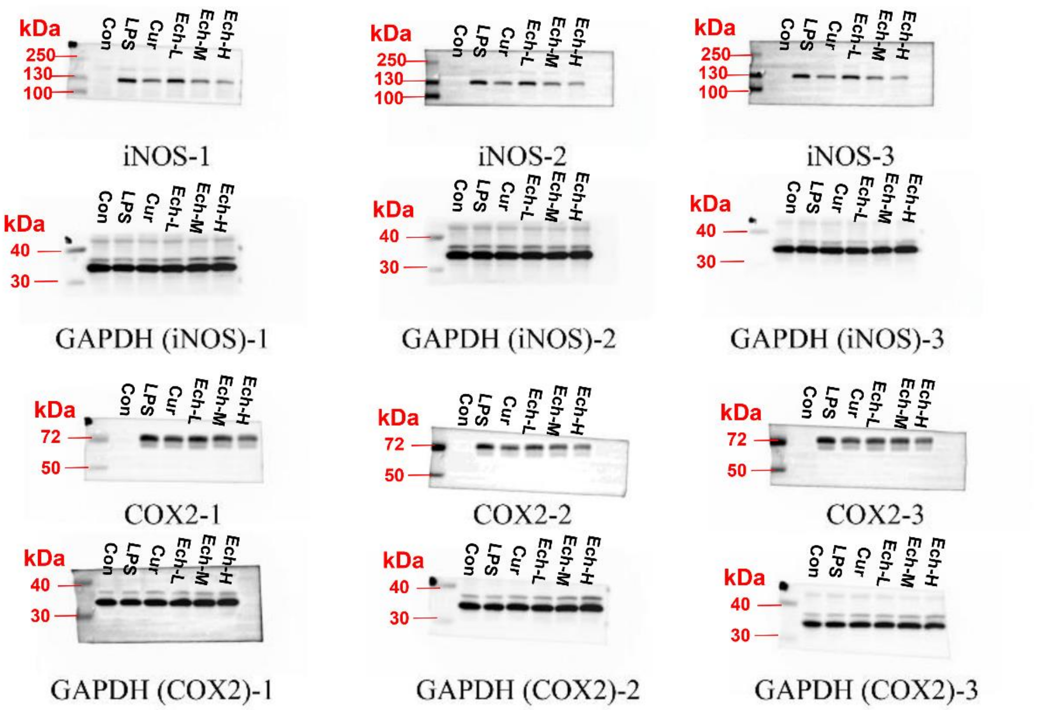

Fig 2

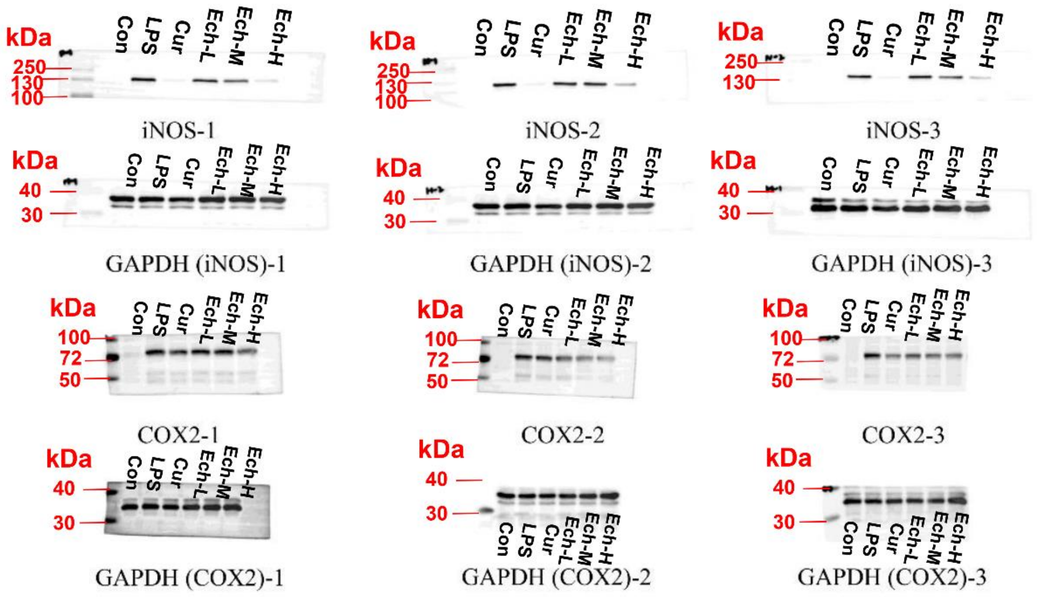
kDa
250
130
100
Con
LPS
Cur
Ech-L
Ech-M
Ech-H
iNOS-1
iNOS-2
iNOS-3
40
30
GAPDH (iNOS)-1
GAPDH (iNOS)-2
GAPDH (iNOS)-3
72
50
COX2-1
COX2-2
COX2-3
GAPDH (COX2)-1
GAPDH (COX2)-2
GAPDH (COX2)-3

Fig 4

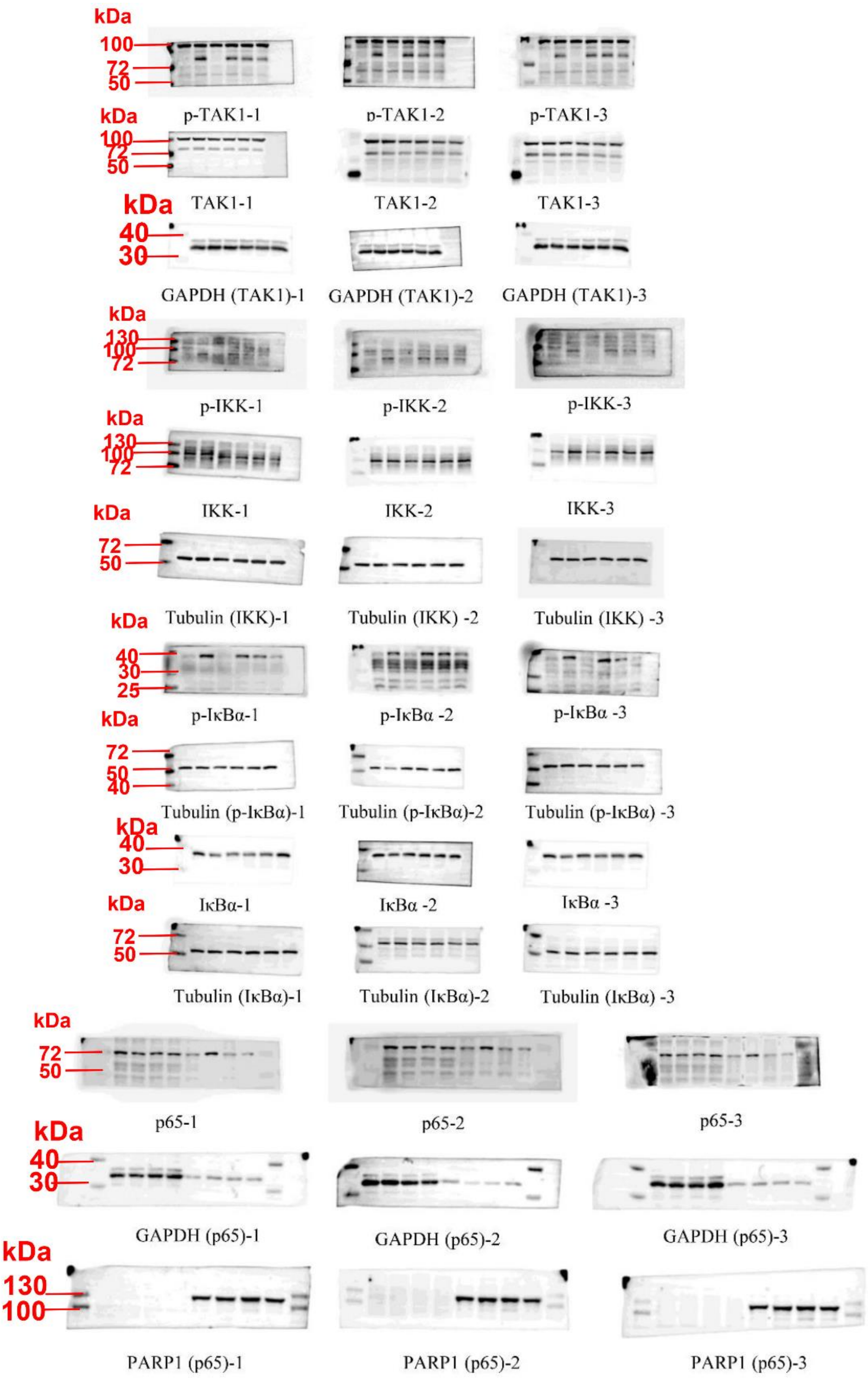
kDa
100
72
50
p-TAK1-1
p-TAK1-2
p-TAK1-3
kDa
100
72
50
TAK1-1
TAK1-2
TAK1-3
kDa
40
30
GAPDH (TAK1)-1
GAPDH (TAK1)-2
GAPDH (TAK1)-3
kDa
130
100
72
p-IKK-1
p-IKK-2
p-IKK-3
kDa
130
100
72
IKK-1
IKK-2
IKK-3
kDa
72
50
Tubulin (IKK)-1
Tubulin (IKK) -2
Tubulin (IKK) -3
kDa
40
30
25
p-IκBα-1
p-IκBα -2
p-IκBα -3
kDa
72
50
40
Tubulin (p-IκBα)-1
Tubulin (p-IκBα)-2
Tubulin (p-IκBα) -3
kDa
40
30
IκBα-1
IκBα -2
IκBα -3
kDa
72
50
Tubulin (IκBα)-1
Tubulin (IκBα)-2
Tubulin (IκBα) -3
kDa
72
50
p65-1
p65-2
p65-3
kDa
40
30
GAPDH (p65)-1
GAPDH (p65)-2
GAPDH (p65)-3
kDa
130
100
PARP1 (p65)-1
PARP1 (p65)-2
PARP1 (p65)-3

Fig 5

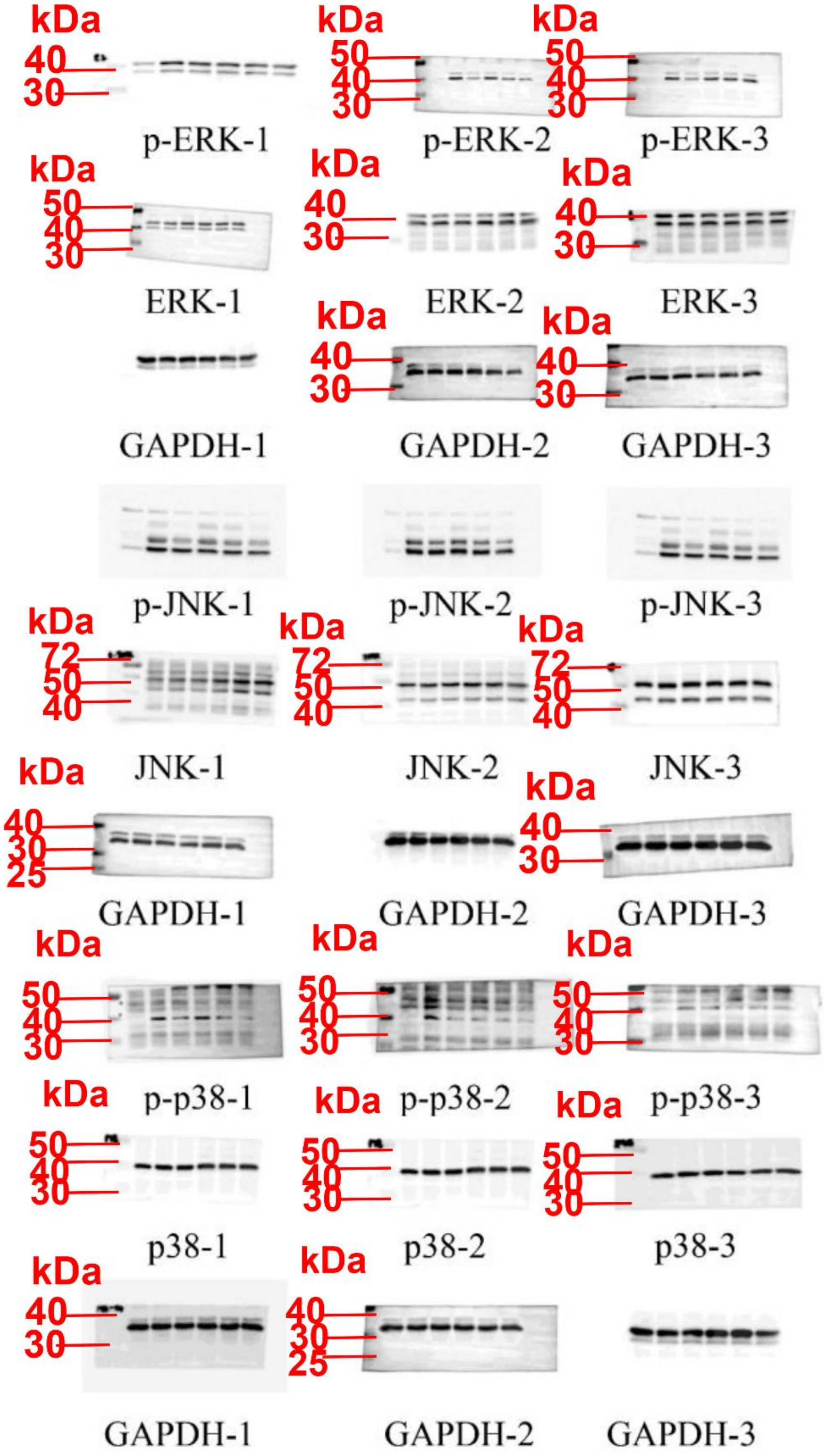
p-ERK-1
p-ERK-2
p-ERK-3
ERK-1
ERK-2
ERK-3
GAPDH-1
GAPDH-2
GAPDH-3
p-JNK-1
p-JNK-2
p-JNK-3
JNK-1
JNK-2
JNK-3
GAPDH-1
GAPDH-2
GAPDH-3
p-p38-1
p-p38-2
p-p38-3
p38-1
p38-2
p38-3
GAPDH-1
GAPDH-2
GAPDH-3
kDa
72
50
40
30
25

Fig 6

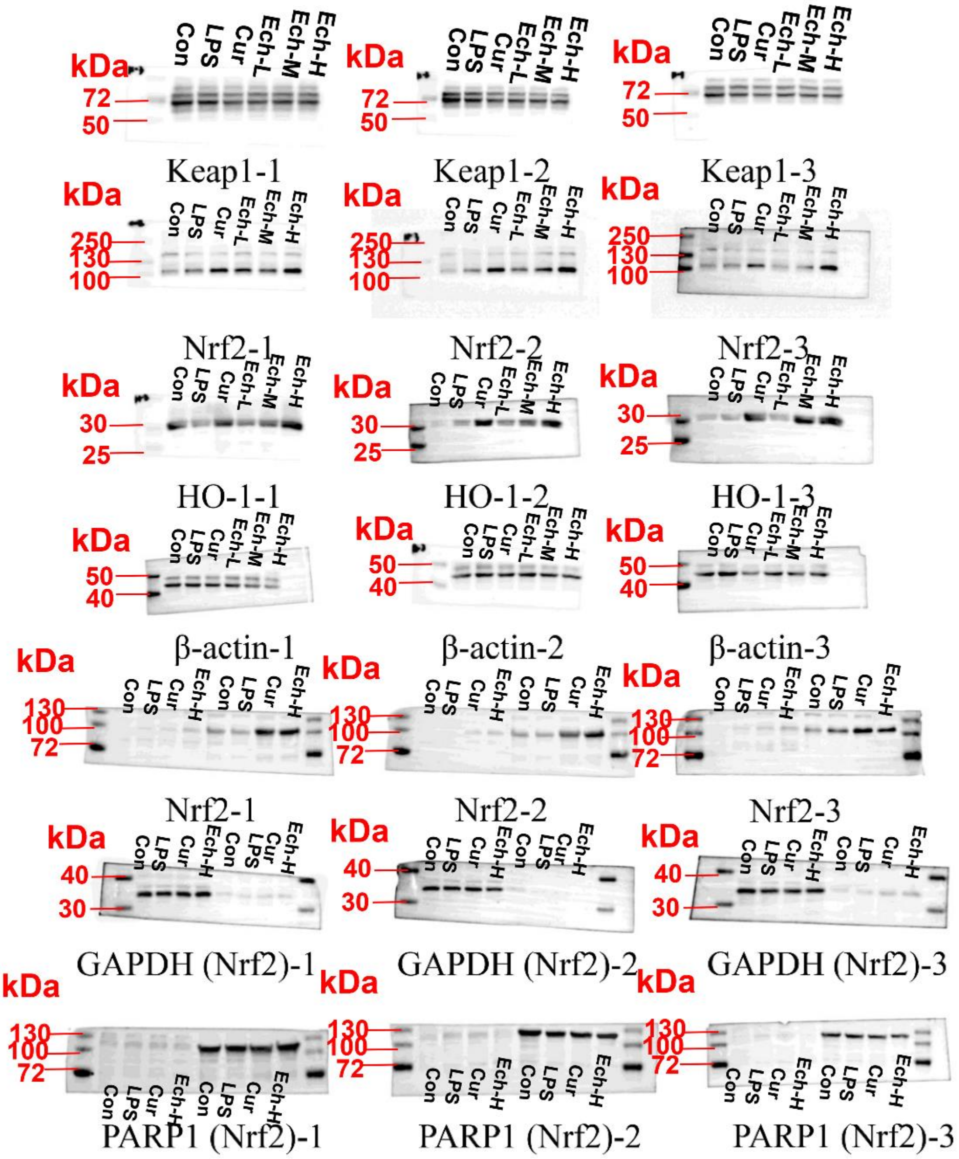
kDa
Con
LPS
Cur
Ech-L
Ech-M
Ech-H
250
130
100
72
50
40
30
25
Keap1-1
Keap1-2
Keap1-3
Nrf2-1
Nrf2-2
Nrf2-3
HO-1-1
HO-1-2
HO-1-3
β-actin-1
β-actin-2
β-actin-3
Nrf2-1
Nrf2-2
Nrf2-3
GAPDH (Nrf2)-1
GAPDH (Nrf2)-2
GAPDH (Nrf2)-3
PARP1 (Nrf2)-1
PARP1 (Nrf2)-2
PARP1 (Nrf2)-3

Fig 7

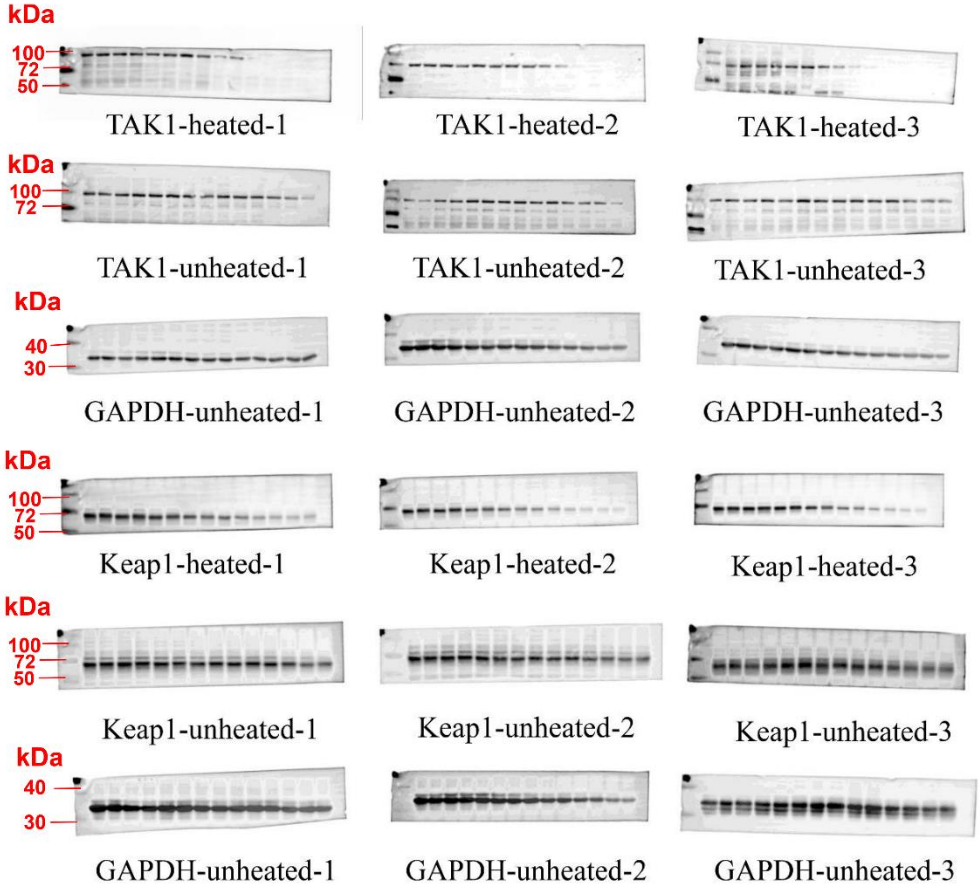

kDa
100
72
50
TAK1-heated-1
TAK1-heated-2
TAK1-heated-3
kDa
100
72
TAK1-unheated-1
TAK1-unheated-2
TAK1-unheated-3
kDa
40
30
GAPDH-unheated-1
GAPDH-unheated-2
GAPDH-unheated-3
kDa
100
72
50
Keap1-heated-1
Keap1-heated-2
Keap1-heated-3
kDa
100
72
50
Keap1-unheated-1
Keap1-unheated-2
Keap1-unheated-3
kDa
40
30
GAPDH-unheated-1
GAPDH-unheated-2
GAPDH-unheated-3

S1 Fig

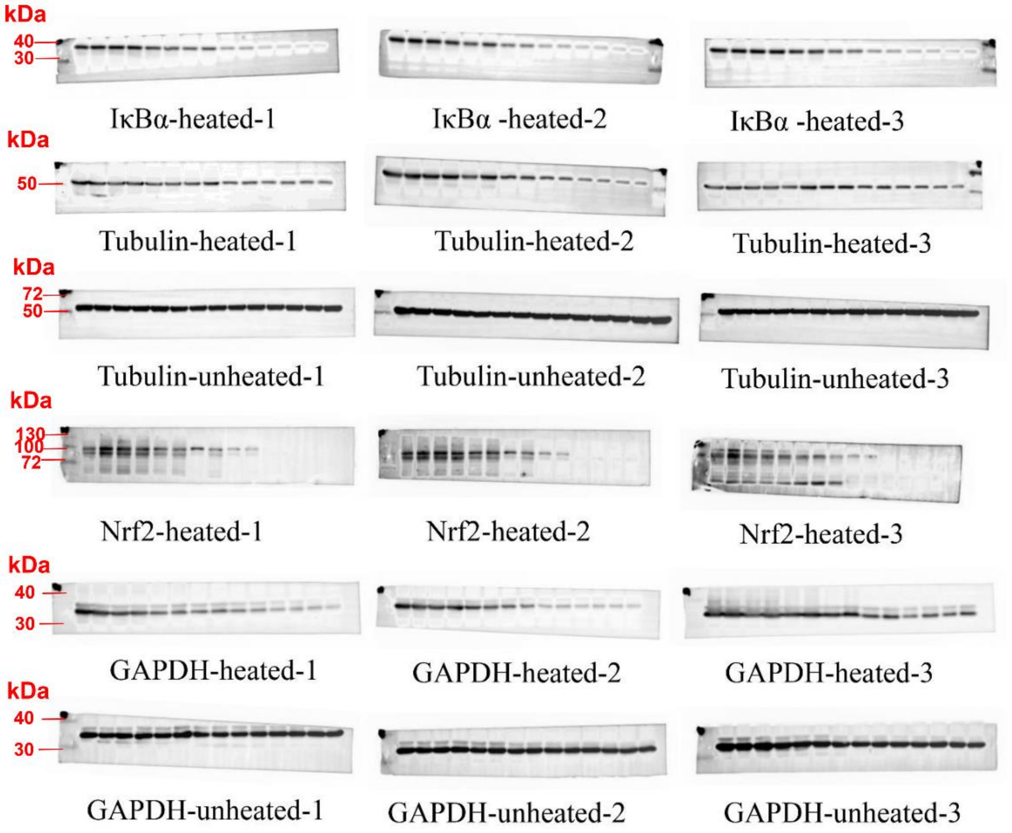
kDa
40
30
IκBα-heated-1
IκBα -heated-2
IκBα -heated-3
kDa
50
Tubulin-heated-1
Tubulin-heated-2
Tubulin-heated-3
kDa
72
50
Tubulin-unheated-1
Tubulin-unheated-2
Tubulin-unheated-3
kDa
130
100
72
Nrf2-heated-1
Nrf2-heated-2
Nrf2-heated-3
kDa
40
30
GAPDH-heated-1
GAPDH-heated-2
GAPDH-heated-3
kDa
40
30
GAPDH-unheated-1
GAPDH-unheated-2
GAPDH-unheated-3
